# Effects of stand age and soil microbial communities on soil respiration throughout the growth cycle of poplar plantations in northeastern China

**DOI:** 10.3389/fmicb.2024.1477571

**Published:** 2024-11-27

**Authors:** Xiangrong Liu, Lingyu Hou, Changjun Ding, Xiaohua Su, Weixi Zhang, Zhongyi Pang, Yanlin Zhang, Qiwu Sun

**Affiliations:** ^1^State Key Laboratory of Tree Genetics and Breeding, Key Laboratory of Tree Breeding and Cultivation of State Forestry Administration, Research Institute of Forestry, Chinese Academy of Forestry, Beijing, China; ^2^State Key Laboratory of Efficient Production of Forest Resource, Research Institute of Forestry, Chinese Academy of Forestry, Beijing, China; ^3^State-Owned Xinmin City Machinery Forest Farm, Shenyang, China

**Keywords:** soil respiration, stand age, soil microbial community structure and function, poplar plantation, microbial r-K selection theory

## Abstract

**Introduction:**

Many studies have identified stand age and soil microbial communities as key factors influencing soil respiration (Rs). However, the effects of stand age on Rs and soil microbial communities throughout the growth cycle of poplar (*Populus euramevicana* cv.‘I-214’) plantations remain unclear.

**Methods:**

In this study, we adopted a spatial approach instead of a temporal one to investigate Rs and soil microbial communities in poplar plantations of 15 different ages (1–15  years old).

**Results:**

The results showed that Rs exhibited clear seasonal dynamics, with the highest rates observed in the first year of stand age (1-year-old). As stand age increased, Rs showed a significant decreasing trend. We further identified r-selected microbial communities (copiotrophic species) as key biological factors influencing the decline in Rs with increasing stand age. Other abiotic factors, such as soil temperature (ST), pH, soil organic carbon (SOC), nitrate nitrogen (NO_3_^−^-N), and the C/N ratio of plant litter (Litter C/N), were also significantly correlated with Rs. Increased stand age promoted fungal community diversity but suppressed bacterial community diversity. Bacterial and fungal communities differed significantly in abundance, composition, and function, with the Litter C/N ratio being a key variable affected by microbial community changes.

**Conclusion:**

This study provides crucial empirical evidence on how stand age affects Rs, highlighting the connection between microbial community assemblages, their trophic strategies, and Rs over the growth cycle of poplar plantations.

## 1 Introduction

Soil respiration (Rs) is the process through which plant roots, fungi, and bacteria in the soil consume organic matter and produce carbon dioxide (CO_2_) (Bond-Lamberty and Thomson, 2010). It is a crucial component of the carbon cycle in terrestrial ecosystems and represents the second largest flux of carbon exchange with the atmosphere (Ma et al., 2023). Rs significantly influences atmospheric CO_2_ concentrations. It has been reported that soil respiration releases 10 times more CO_2_ into the atmosphere annually than fossil fuel combustion (Masson-Delmotte et al., 2021). Consequently, even small changes in Rs can substantially impact atmospheric CO_2_ levels and the global terrestrial ecosystem carbon cycle (Feng et al., 2018). Since the industrial revolution, atmospheric CO_2_ concentrations have surged, leading to a rise in global temperatures. In the context of global warming, understanding the characteristics of Rs and the factors influencing it is vital for estimating changes in atmospheric CO_2_ concentrations. This understanding is crucial for accurately assessing regional carbon balances (Huang et al., 2020; Ma et al., 2014).

In terrestrial ecosystems, forests act as important natural carbon sinks and have a negative feedback effect on global warming (Bonan, 2008; Pan et al., 2011). Forests comprise roughly half of the biomass in terrestrial ecosystems and exert a significant and indispensable influence on the global soil carbon pool (Dixon et al., 1994). Stand age is a crucial indicator of forest developmental succession and carbon dynamics, with significant amounts of organic matter accumulating in the surface layer of forest soils over time (Bastida et al., 2019; Tang et al., 2009). Whether forest ecosystems act as carbon sources or sinks largely depends on the balance between photosynthetic carbon sequestration and respiration. Consequently, many studies have focused on the mechanisms of Rs changes and the factors influencing them. Previous studies on the impact of stand age on Rs has yielded three primary findings. First, Rs increases with stand age, mainly due to the growth and accumulation of soil organic carbon (SOC), roots, and microbial biomass (Wiseman and Seiler, 2004; Xiao et al., 2014). Second, there is an inverse relationship between Rs and stand age, attributed primarily to the decline in fine root biomass and metabolic activity (Gong et al., 2012; Yan et al., 2011; Zhao et al., 2016). Finally, some studies have found no significant linear correlation between Rs and stand age (Powers et al., 2018; Tang et al., 2009). The previous studies have selected discontinuous stand ages, and has not focused throughout the growth cycle.

Afforestation can increase carbon sequestration in terrestrial ecosystems, mitigate soil erosion, and reduce greenhouse gas emissions (Liu et al., 2013; Reay et al., 2007). Ecological restoration projects implemented in China in the late 1970s significantly contributed to carbon emissions in the 2001–2010 decade. These contributions were equivalent to 9.4 percent of the carbon emissions from fossil fuels during the same period (Fang et al., 2018; Lu et al., 2018). China’s poplar plantation area reaches 7.57 Mha, ranking first in the world (National Forestry and Grassland Administration, 2019). Poplar is widely planted as pure or mixed species plantations, which has an economic value in providing wood and energy raw materials (Pilipovic et al., 2022), as well as mitigating the problem of dust storms and sandstorms in spring in northern China, which is of great significance for ecological environment protection and restoration of the areas in need of windbreaks and sand fixation. The increase in the area of poplar plantations is also due to their strong adaptability, rapid growth, and outstanding advantages in carbon sequestration (Gielen and Ceulemans, 2001). Additionally, poplars are also one of the major emitters of isoprene, a volatile organic compound (VOC) naturally released by trees, which has a significant impact on the atmospheric carbon cycle. Furthermore, the ability of poplars to emit isoprene is influenced by physiological states such as developmental stages, and is regulated by environmental factors such as temperature, light intensity, nitrogen nutrition, and atmospheric CO_2_ concentration (Teuber et al., 2007). Therefore, the study of the relationship between the age of poplar plantations and carbon emissions is of great significance in addressing climate change and assessing carbon balance.. However, the relationship between Rs and throughout the growth cycle of poplar plantation has not been studied yet.

Previous research in forest ecosystems has shown that Rs is correlated with various factors, including soil temperature and moisture (ST and SM), soil physicochemical properties, and forest type (Zhao et al., 2016). In recent years, many studies have analysed microbial communities properties and carbon cycle which could improve the prediction of Rs (Liu et al., 2023; Nottingham et al., 2022; Zeng et al., 2022; Zhang and Zhang, 2016). Research has shown that Rs is correlated with soil microbial communities, particularly with Alphaproteobacteria, Acidobacteria, and Basidiomycota (Chen et al., 2021b). Additionally, in subtropical subalpine mountain ecosystems, it has been discovered that bacterial communities have a significant positive correlation with Rs. Rare bacterial phyla (e.g., Gemmatimonadetes and Cyanobacteria) are the primary driving factor, exerting a greater influence on Rs than abundant microbial communities (Han and Wang, 2023). According to the microbial trophic utilization patterns and carbon mineralization characteristics, microbial communities are usually divided into two ecological functions: r-strategists (copiotrophic) and K-strategists (oligotrophic) (Fierer et al., 2007). R-selected species grow fast and often utilize labile carbon. In contrast, k-selected species grow slowly and often utilize recalcitrant carbon. Fungi tend to grow slower than bacteria and are often classified as k-strategists (Yang et al., 2022). Related studies have reported specific roles for both copiotrophs and oligotrophs bacteria in utilizing carbon for respiration, and that the role of copiotrophs bacteria is higher than that of oligotrophs bacteria (Liu et al., 2020; Liu et al., 2018a; Liu et al., 2020b; Siles and Margesin, 2016). In summary, these findings indicate a close relationship between microbial communities and Rs.

In this study, we applied a spatial - for - temporal substitution method, focusing for the first time on poplar plantations in the Songliao Plain, Northeast China, spanning 1 to 15 years. We analyzed the link between stand age and Rs during continuous growth. This approach aims to explore the impact of stand age on Rs, providing new theoretical perspectives and empirical evidence for related research. The influence of stand age on soil respiration (Rs) was investigated from July 2022 to June 2023. The key objectives were to delve into (1) the pattern of change in Rs with stand age; (2) the dynamics of soil microbial communities with stand age; and (3) the relationship between biotic and abiotic factors and Rs.

## 2 Materials and methods

### 2.1 Site description and experimental design

The study site is located in a forest farm in Xinmin City, Liaoning Province, China (41°42′–42°17′N, 122°27′–123°20′E) (Supplementary Figure S1). The area belongs to the sandstorm area in the northern part of the Liaohe Plain, with a low terrain, gradually higher from south to north, and an average elevation of 29 metres above sea level. The study site experienced a temperate continental monsoon climate, with mean annual temperatures and precipitation of 8.2°C and 417.7 mm, respectively. It had an average frost-free period of 160 days and an annual sunshine duration averaging 2753.2 h. The soil type is brown soil.

In June 2022, based on principles such as site conditions and consistent species, we selected 15 stand ages ranging from 1 to 15 years as the research objects. In each forest area, we randomly selected three 20 m × 20 m plots with similar growth as independent replicates, with the plots being no more than 30 meters apart from each other, establishing a total of 45 plots. All sampled plots were designated as forest land, with all previously being managed as pure artificial forests of the same species of poplar trees and subjected to identical management practices. To minimize the differences in climate, topography, and other factors among the plots, ensuring that variations in soil respiration and soil properties are attributed solely to stand age, the distance between any two stands was kept to no more than 3 km.

In October 2022, after removing surface litter, we collected 10 soil cores from each plot at a depth of 0 - 20 cm using the “S” sampling method and combined them into a single composite sample per plot. First, stones and plant roots were removed from the samples, which were then passed through 2 mm and 0.149 mm mesh sieves. The soil samples designated for physicochemical property analysis were air-dried and stored. Meanwhile, the soil samples intended for microbial community analysis were stored at - 80°C. Plant leaf litter was dried to a constant weight and then ground into powder for later use.

### 2.2 Field measurement of Rs rates

In June 2022, six PVC soil rings were randomly installed within each stand-age sample for Rs measurements. The PVC ring has a diameter of 20 cm and a length of 15 cm, with about 5 cm exposed above the soil surface, and remains in place throughout the entire experimental process. Before each measurement, plants within the soil ring were removed, along with the litter, to avoid the influence of plants and litter on Rs. Measurements were taken using the Li-8100a automated soil CO_2_ flux system (LI-COR Inc., Lincoln, NE, United States) in the middle and end of each month from July 2022 to June 2023, respectively (no measurements were taken in December–February due to snow cover that froze the soil), with each measurement taken between 8.00 a.m. and 11.30 a.m. While measuring Rs rates, ST and SM at a depth of 5 cm in the vicinity of the soil ring were also measured.

### 2.3 Analysis of soil and litter samples

The soil bulk density (SBD) was measured using the cutting ring method (Wang et al., 2018). Soil pH was measured by using the soil-water (1:2.5) mixed suspension through the potential method. Soil and litter organic carbon was determined by the K_2_CrO_7_ oxidation method (Lefroy et al., 1993). Total soil and litter nitrogen was determined by Kjeldahl method. SOC and TN data were used to calculate soil C/N. Litter C/N was calculated using litter organic carbon and total nitrogen data. Dissolved organic carbon (DOC) was extracted with deionized water (1:4) and then measured using a TOC analyzer (Shimadzu Corp, Kyoto, Japan). Easily oxidised organic carbon (EOC) was determined after KMnO_4_ oxidation (Zhao et al., 2018). The available phosphorus (AP) and available potassium (AK) were extracted from the soil samples using a 2% (NH_4_)_2_ CO_3_ solution at a soil-to-solution ratio of 1:5, and were subsequently determined using an ICAP instrument (Spectro Analytical Instruments, Spectro Arcos ICP, Kleve, Germany) (Wang et al., 2022). Nitrate nitrogen (NO_3_^−^-N) and ammonium nitrogen (NH_4_^+^-N) were extracted from the soil using 2 M KCl and the content was determined using a continuous flow injection analyser system (AA3 Continuous Flow Analytical System).

### 2.4 Soil DNA extraction and sequence analysis

Soil total DNA was extracted from 0.25–0.5 g of soil samples using the TIANNamp Guide S96 kit (Beijing, China). The extracted nucleic acids were assayed for concentration using an enzyme labeler (GeneCompang Limited, synergy HTX). After passing the assay, the nucleic acids were amplified, and the amplified PCR products were then detected by electrophoresis using agarose at a concentration of 1.8%. The bacterial V3–V4 region was amplified using the 16S rRNA gene primers 338F (5′-ACTCCTACGGGAGGCAGCAG-3′) and 806R (5′-GGACTACHVGGGTWTCTAAT-3′). The fungal ITS1 region was amplified using the fungal ITS primers ITS1F (5′-CTTGGTCATTTAGAGGAAGTAA-3′) and ITS2R (5′-GCTGCGTTCTTCATCGATGC-3′). The products were purified, quantified, and normalized on the Illumina Novaseq 6000 platform, and detected using the NovaSeq 6000 S4 Reagent Kit (San Diego). The original image data files obtained from sequencing are converted into raw sequencing sequences through base recognition analysis. The sequencing results are filtered, and then primer sequences are identified and removed to obtain effective sequences.

The soil microbial communities (bacteria and fungi) were classified as r-strategists or K-strategists based on their nutrient utilization modes. Among them, copiotrophic bacteria, such as Actinobacteriota, Bacteroidota, Firmicutes, and Gemmatimonadota, were categorized as r-strategists. The oligotrophs bacterial members, including Acidobacteriota, Chloroflexi to K-strategists. The r-strategist fungi include Ascomycota, while the K-strategist fungi include Basidiomycota (Li et al., 2021; Fierer et al., 2007; Yang et al., 2022). We also selected several microbial taxa associated with the carbon cycle, including Proteobacteria, Acidobacteriota, Actinobacteriota, Bacteroidota, Ascomycota, and Basidiomycota. We analyzed their relative abundance to investigate the potential relationship with Rs (Chen et al., 2021b; Castro et al., 2019).

### 2.5 Statistical analysis

Regression analyses were used to evaluate the relationship between soil properties, microbial community structure, characteristics and stand age. Pearson correlation analysis was used to determine the relationship between Rs and biotic and abiotic factors. Using the “plspm” package (Sanchez et al., 2013), the effects of stand age, climate (ST, SM), soil and plant properties (pH, SOC, DOC/SOC, EOC/SOC, AK, NO_3_^−^-N, and litter C/N), as well as soil bacterial and fungal community structure, on Rs were evaluated through a partial least squares path model (PLS-PM). One-way analysis of variance (ANOVA) was used to compare the dominant communities of soil bacteria and fungi in stands of different stand ages. Differences in Bray–Curtis distances between stand ages for bacterial and fungal communities were analysed using non-metric multidimensional scaling (NMDS); the significance of the differences was determined by using ranked analysis of variance (PERMANOVA). The relationship between soil and plant properties and soil bacterial and fungal diversity and community structure was analysed using the Mantel test. In addition, functional information was annotated for OTU of bacteria and fungi using FAPROTAX and FUNGuild software (Louca et al., 2016; Nguyen et al., 2016). All data analysis and figures were conducted using SPSS18.0 (IBM, United States), Origin2021 (OriginLab, United States), and R software (v4.3.2).

## 3 Results

### 3.1 Changes in Rs with stand age Changes and relationships between Rs and soil factors under stand age effects

**Figure 1:**
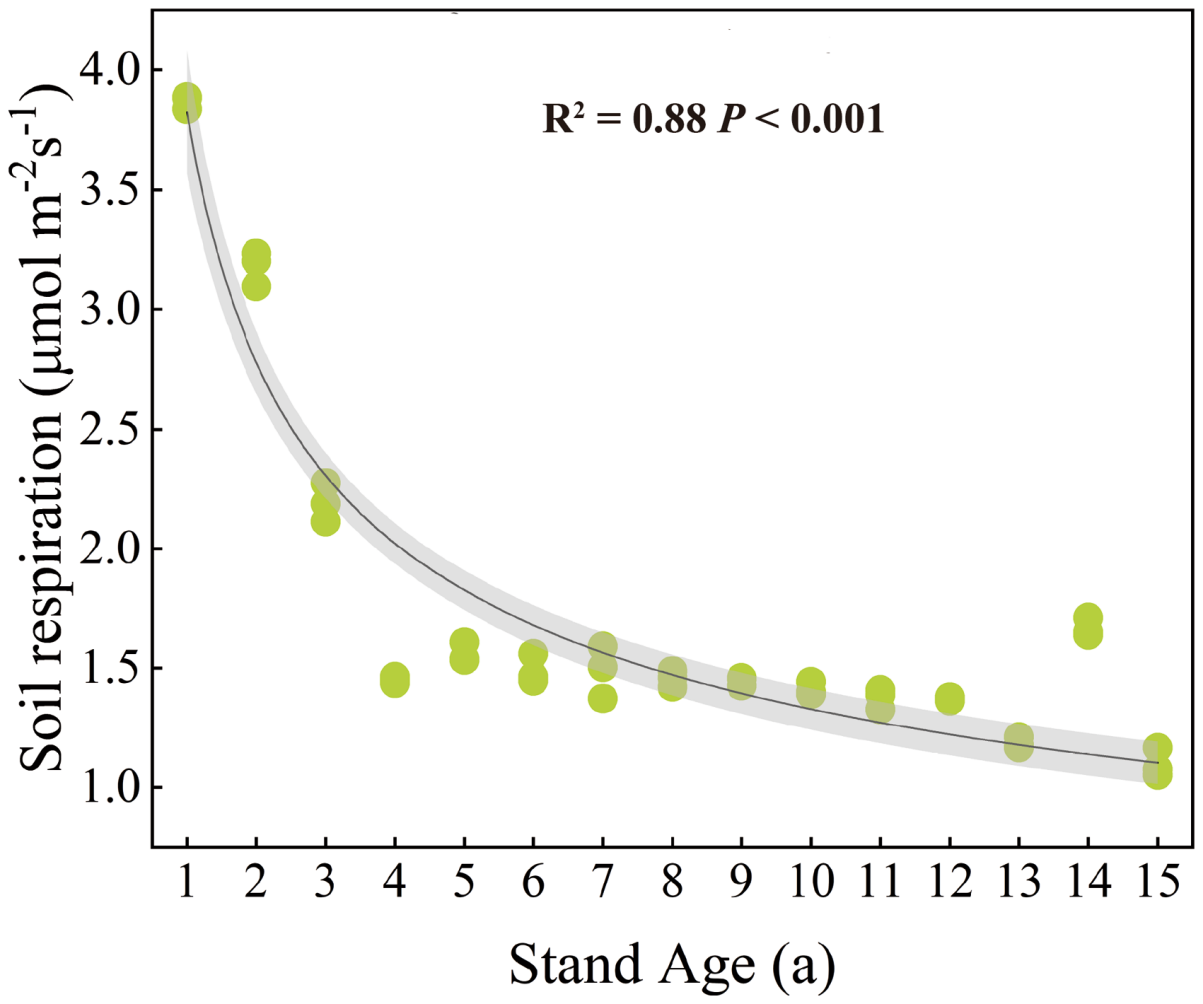
Patterns of change in soil respiration with stand age.

Rs rate significantly decreased with increasing stand age (*p* < 0.001) (Figure 1). SBD, SOC, DOC, and NO_3_^−^-N showed significant changes with stand age (*p* < 0.05) (Supplementary Figure S2). Among these, SBD and DOC generally increased with stand age, while SOC and NO_3_^−^-N decreased. All factors except for soil pH, DOC, NH_4_^+^-N, and AP were significantly correlated with Rs rate (*p* < 0.05) (Supplementary Figure S3). Specifically, SOC, TN, NO_3_^−^-N, and AK were significantly positively correlated with Rs, while SBD and EOC were significantly negatively correlated. Rs rate was significantly negatively correlated with both the DOC/SOC and EOC/SOC, and significantly positively correlated with litter C/N (*p* < 0.05), but showed no significant correlation with soil C/N (Supplementary Figure S4).

### 3.2 Soil bacterial community composition and its relationship with environmental factors

**Figure 2:**
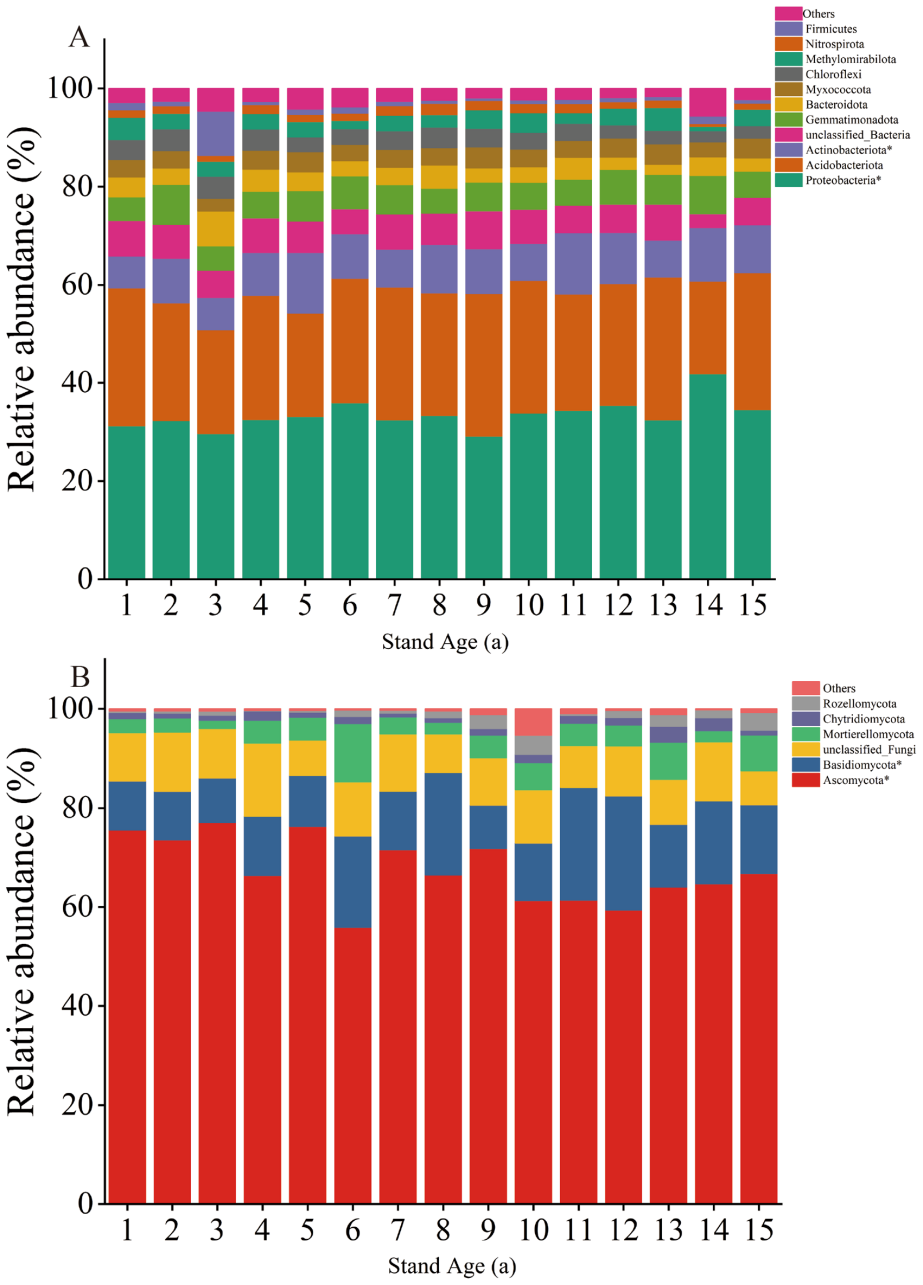
Relative abundance of dominant bacterial **(A)** and fungal **(B)** groups at the phylum level. ^*^Significant differences (*p* < 0.05) were found between different stand ages.

The bacterial community structure and diversity changed with increasing stand age. The dominant phyla across different stand ages were Proteobacteria (29.19–41.88%), Acidobacteriota (18.90–29.11%), and Actinobacteriota (6.49–12.50%), comprising 71.96–79.34% of the bacterial samples at each stand age. The relative abundance of these dominant phyla significantly changed with stand age. Notably, the relative abundance of Proteobacteria and Actinobacteriota showed a significant increasing trend with stand age (*p* < 0.05) (Figure 2A).

Functional annotation of bacterial OTUs using FAPROTAX was used to identify carbon and nitrogen transforming functional bacterial groups in poplar associated soils and to determine their relative abundance in each stand. Related to the carbon cycle are phototrophy, photoautotrophy and oxygenic photoautotrophy. Associated with the nitrogen cycle are nitrate reduction and nitrogen fixation. The results indicate that as the stands mature, there is a general decrease in the abundance of nitrate-reducing bacteria involved in nitrogen transformation, while nitrogen-fixing bacteria show an increasing trend (Supplementary Figure S5A). Concurrently, the abundance of photoautotrophic organisms involved in carbon transformation tends to decrease with stand age (Supplementary Figure S5A).

**Figure 3:**
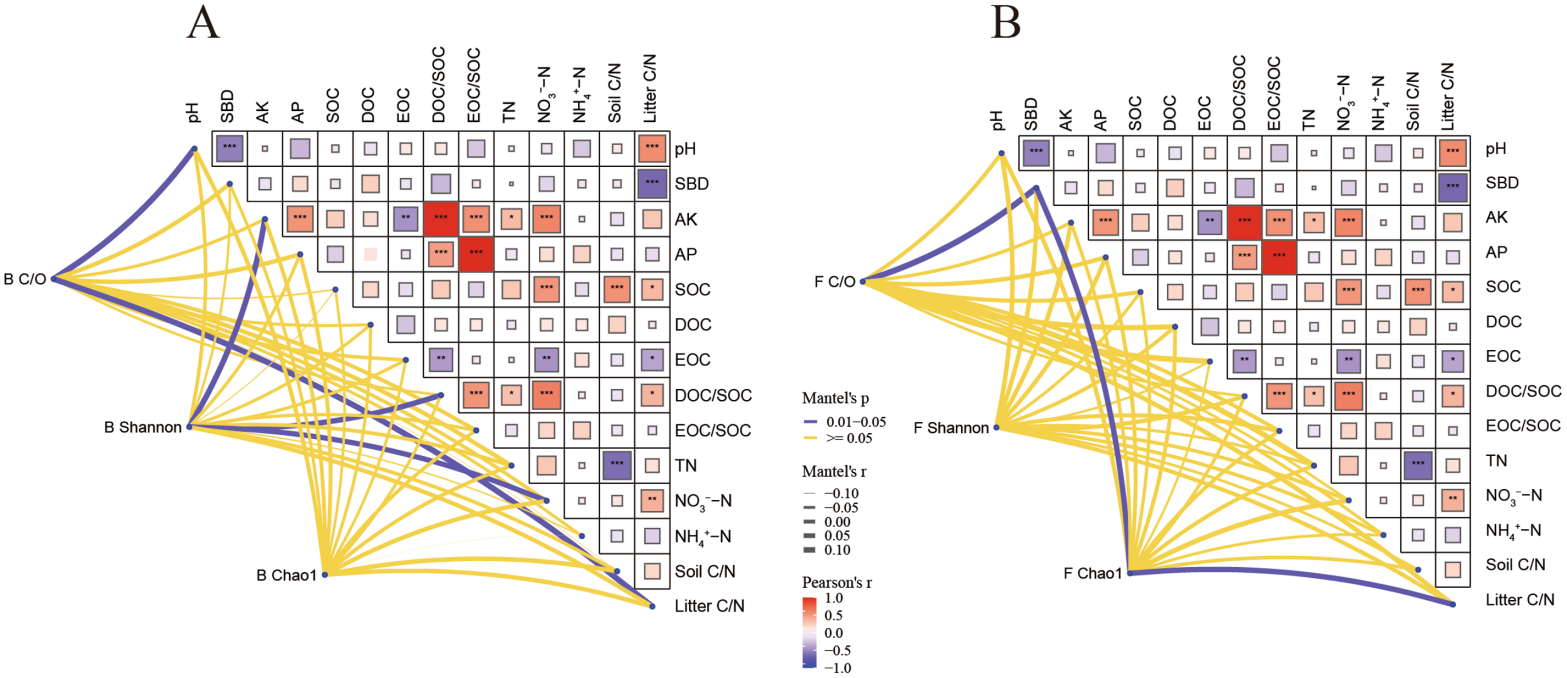
The Mantel test of the correlations analysed the relationship between soil and plant characteristics and soil bacterial **(A)** and fungal **(B)** diversity and community structure. B C/O, bacterial copiotroph/oligotroph ratio; B Shannon, bacteria Shannon; B Chao1, bacteria Chao1; F C/O, fungal copiotroph/oligotroph ratio, F Shannon, fungi Shannon; F Chao1, fungi Chao1; SBD, soil bulk density; AK, available potassium; AP, available phosphorus SOC, soil organic carbon; DOC, dissolved organic carbon; EOC, easily oxidised organic carbon; TN, total nitrogen; NO_3_^−^-N, nitrate nitrogen; NH_4_^+^-N, ammonium nitrogen; litter C/N, plant litter C/N. In the figure, the heatmap colors and grid sizes are simultaneously used to map the magnitude of correlation values. Darker colors and larger grid sizes indicate larger absolute values of correlation, while lighter colors and smaller grid sizes indicate smaller absolute values of correlation. Colors closer to red indicate stronger positive correlation, while colors closer to blue indicate stronger negative correlation. The marks within the grids, *p < 0.05; **p < 0.01; ***p < 0.001, indicate statistically significant results. The thickness of the lines connecting the three nodes with various soil and plant factors serves as an indication of the magnitude of their correlation. Thicker lines signify stronger correlations, whereas thinner lines indicate weaker correlations. Blue lines represent significant correlations (*p* < 0.05), while yellow lines denote non-significant correlations (*p* > 0.05).

Microbial characteristics changed significantly with stand age (*p* < 0.05) (Supplementary Figure S6). Bacteria Shannon decreased significantly with stand age and bacterial NMDS1 increased significantly with stand age. Bacterial to fungal ratio Shannon, on the other hand, decreased significantly with stand age. The results of Mantel test showed that pH, litter C/N, AK, DOC/SOC and NO_3_^−^-N were important factors affecting the structure of bacterial community (*p* < 0.05) (Figure 3A). MMDS analyses showed that bacterial community β-diversity varied considerably with stand age, and bacterial community composition was significantly segregated between different stand ages (*p* < 0.05; Supplementary Figure S7A). PERMANOVA analyses further determined significant differences in bacterial (*p* = 0.001) community composition across the 15 stand ages.

### 3.3 Soil fungal community composition and its relationship with environmental factors

The primary fungal phyla were Ascomycota (55.92–77.02%) and Basidiomycota (8.83–23.08%), collectively comprising 72.88–87.17% of fungal sequences in each sample, with Ascomycota predominating. The relative abundance of these fungal communities was significantly influenced by stand age. Ascomycota exhibited an overall significant decreasing trend, while Basidiomycota showed a significant overall increasing trend (*p* < 0.05) (Figure 2B).

The FUNGuild database was used to predict the functional attributes of fungal communities, firstly by classifying fungi into three main groups based on the mode of nutrition: saprotroph, symbiotroph and pathotroph. Based on the predicted results, the saprotroph types include dung saprotroph, litter saprotroph, plant saprotroph, soil saprotroph, undefined saprotroph and wood saprotroph. Symbiotroph trophic types include ectomycorrhizal and endophyte. Pathotroph types include plant pathogen, animal pathogen and fungal parasite. Stand age significantly influenced the abundance of fungal functional communities. It is noteworthy that dung saprotroph and litter saprotroph abundance decreased significantly with stand age (*p* < 0.05) (Supplementary Figure S5B).

Fungal microbiological properties changed significantly with stand age (*p* < 0.05) (Supplementary Figure S6). Fungi Shannon increased significantly with stand age and fungal NMDS1 increased significantly with stand age. The Mantel test revealed a significant correlation between litter C/N and fungal Chao1, and highlighted SBD as a key determinant influencing fungal community structure (*p* < 0.05) (Figure 3B). NMDS analyses showed that the β-diversity of fungal communities varied considerably with stand age, and the composition of fungal communities was significantly segregated between different stand ages (*p* < 0.05) (Supplementary Figure S7B). PERMANOVA analyses further determined significant differences in fungal community composition across the 15 stand ages (*p* = 0.032).

### 3.4 Characterisation of Rs dynamics and its relationship with biotic and abiotic factors

**Figure 4:**
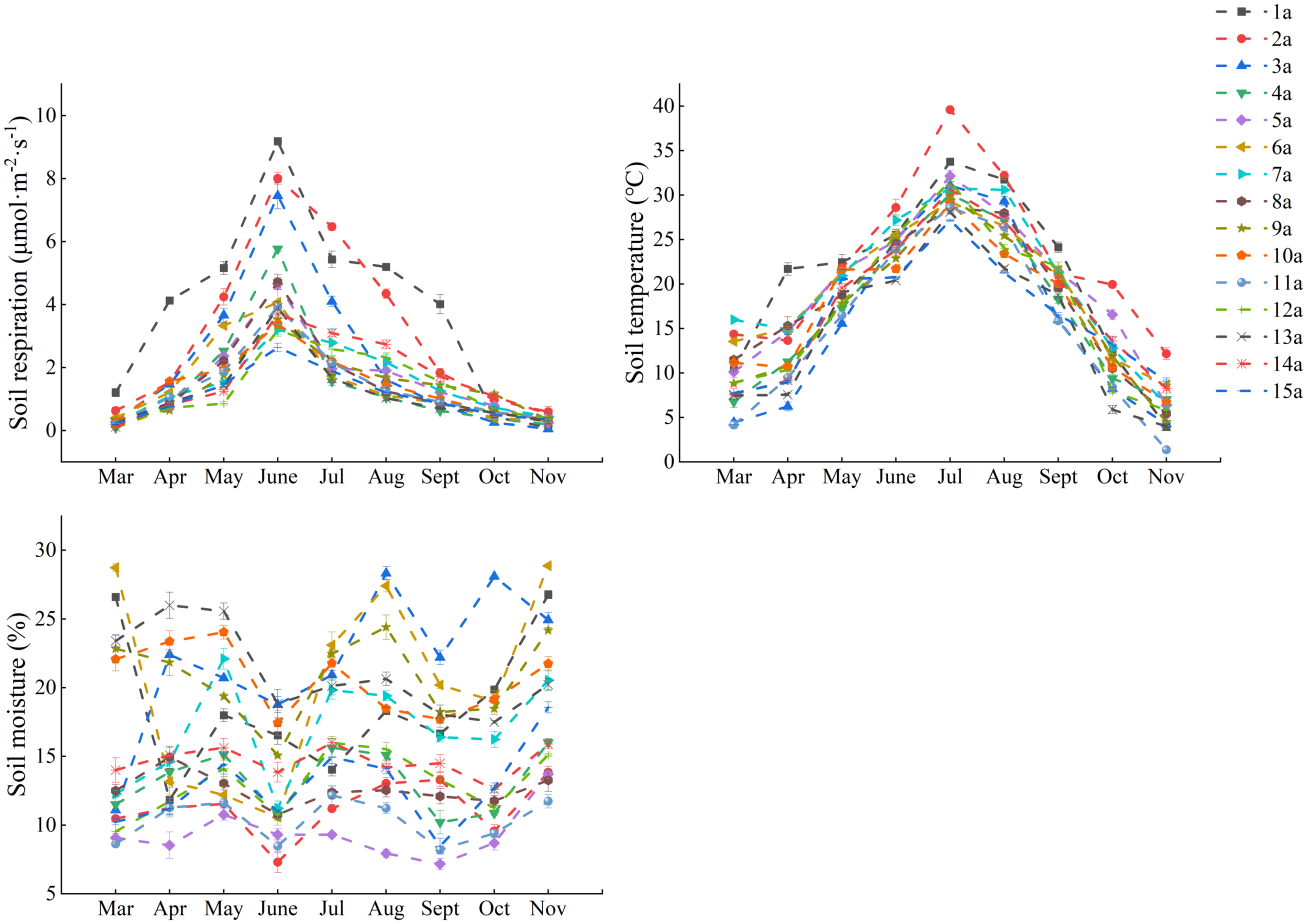
Seasonal changes in soil respiration, soil temperature and soil moisture in 15 poplar plantations of different stand ages.

Overall, Rs rates of all stand ages showed similar seasonal variations over the observation period. Rs varied markedly by stand age, with Rs rate being greatest in 1a and showing an overall decreased trend with increased stand age. ST changes were consistent with the dynamics of Rs rate, while SM dynamics were inconsistent with the pattern of Rs rate and ST changes (Figure 4).

**Figure 5:**
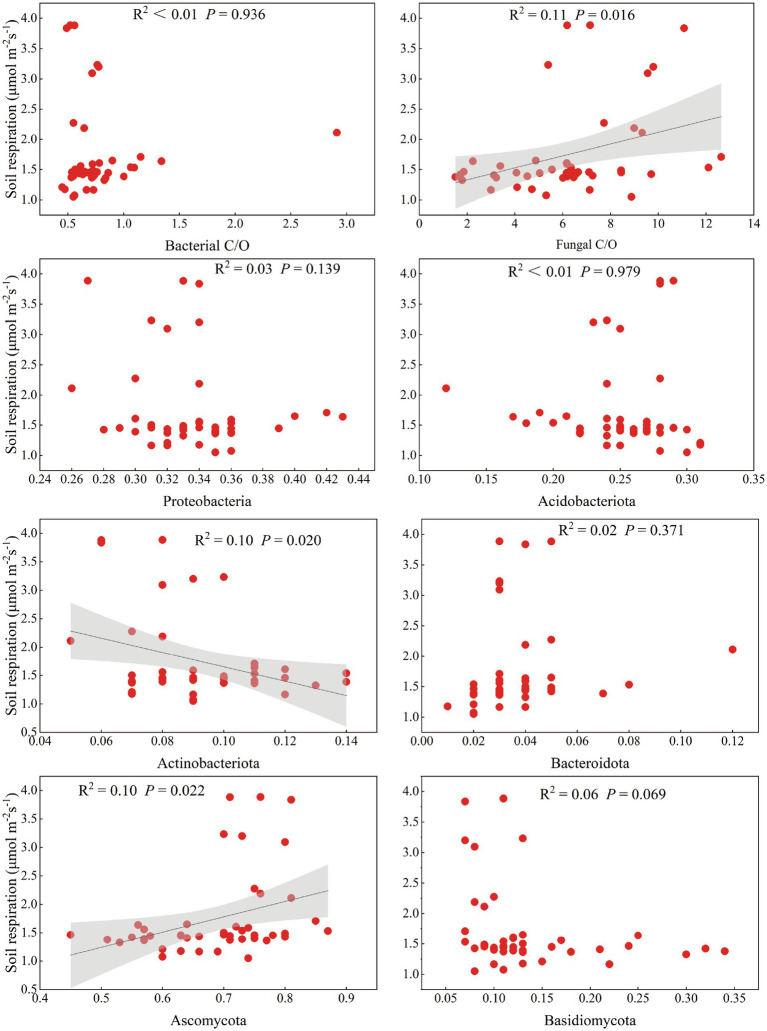
Relationship between soil respiration rate and relative abundance of major microbial taxa associated with carbon mineralisation. The solid black line indicates a significant linear relationship consistent with the regression model, and the shaded area indicates the fitted 95% confidence interval.

Pearson correlation analysis revealed that Rs was significantly correlated with stand age, SBD, ST, DOC/SOC, EOC/SOC, AK, TN, NO_3_^−^-N, and litter C/N (*p* < 0.05) (Supplementary Figure S8). To further explore the relationship between microbial diversity and Rs, we found that the shift in microbial diversity had a significant impact on Rs as the poplar plantations matured (Supplementary Figure S9). For example, changes in the relative abundance of specific microbial groups, such as Actinobacteria and Ascomycota, might alter the decomposition rate of organic matter, thereby affecting Rs (Figure 5). In addition, the balance between bacterial and fungal communities also played a crucial role in regulating Rs. This is consistent with the correlation between the bacterial Shannon index and Rs, indicating that higher bacterial diversity contributes to more efficient carbon cycling at younger stand ages.

**Figure 6:**
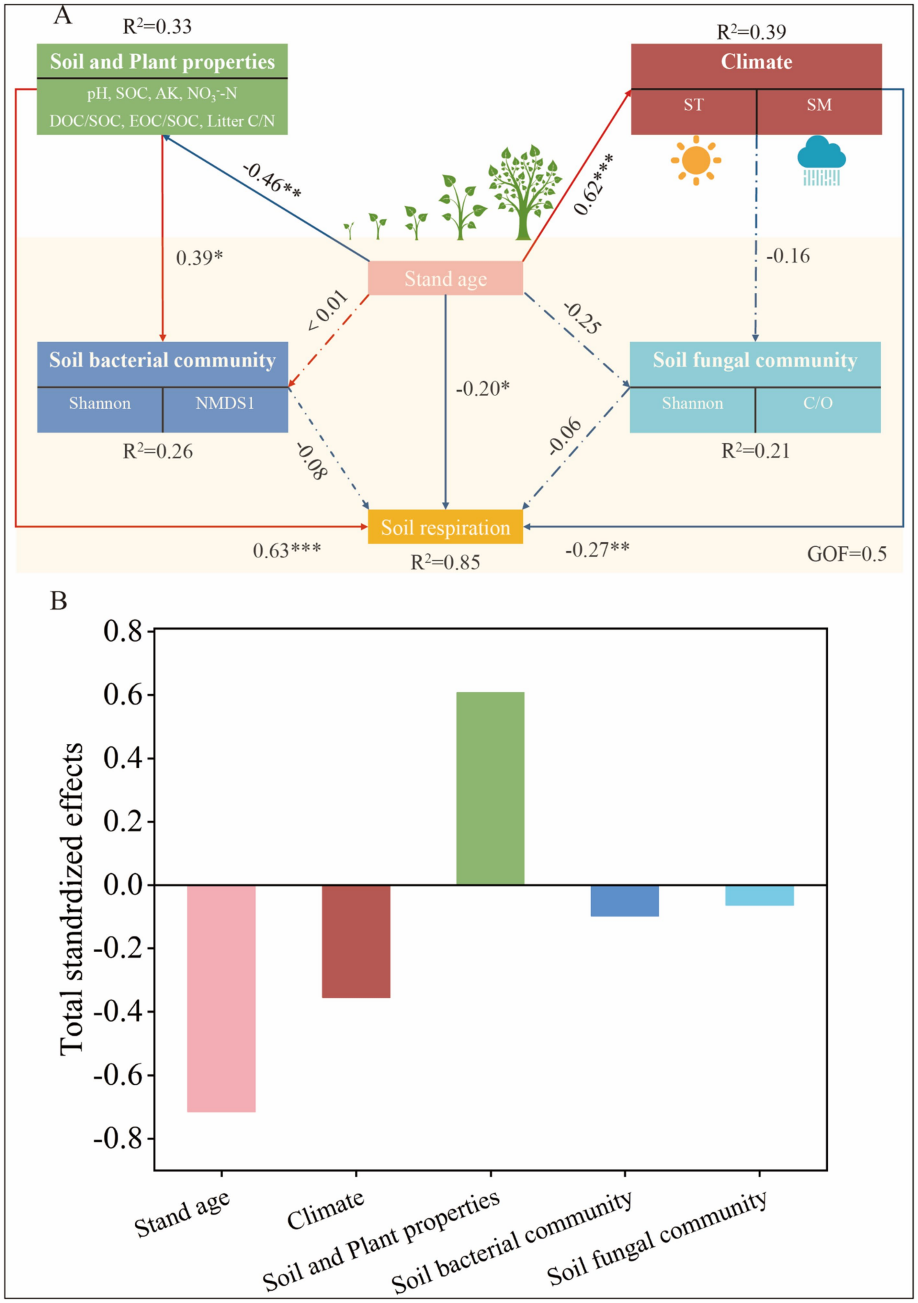
Partial least squares path modelling (PLS-PM) of soil respiration by stand age, climate, soil properties, soil bacterial and fungal communities **(A)**, and the standardised total effects of soil respiration influences **(B)**. Causal relationships are indicated by arrows and the numbers next to the arrows indicate the standardised path coefficients. Solid lines indicate significant relationships (^*^p < 0.05, ^**^p < 0.01, and ^***^p < 0.001) and dashed lines indicate non-significant relationships. *R*^2^ denotes the variance of the variables considered in the model. GOF, goodness of fit. SOC, soil organic carbon; AK, available potassium; NO_3_^−^-N, nitrate nitrogen; EOC, Easily oxidised organic carbon; Litter C/N, plant litter C/N; ST, soil temperature; SM, soil moisture; Shannon, diversity indices of bacterial and fungal communities; C/O, copiotroph/oligotroph ratio.

We further analyzed the possible links between stand age, soil properties, climate, soil microbial (bacterial and fungal) community structure, and Rs using PLS-PM, as well as the direct and indirect effects they produce (Figure 6). bove all, stand age and soil properties accounted for the greatest proportion of variation in Rs (Figure 6B). Stand age had a significant indirect effect on soil bacterial community structure (path coefficient: −0.46) (*p* < 0.01), suggesting that as poplar plantations mature, shifts in microbial diversity may contribute to reduced Rs rates (path coefficient: −0.45) (*p* < 0.05). Changes in soil bacterial community structure were also directly determined by soil properties (path coefficient: 0.39). Stand age indirectly and significantly influenced Rs through soil properties (path coefficient: −0.46) (*p* < 0.005) and climate (path coefficient: 0.62) (*p* < 0.01), while changes in Rs were also directly influenced by stand age (path coefficient: −0.20), soil properties (path coefficient: 0.63), and climate (path coefficient: −0.27), respectively. Overall these variables explained 85% of the variation in Rs.

## 4 Discussion

### 4.1 Relationship between stand age and Rs

There are no existing studies that investigate the impact of stand age on Rs throughout the entire growth cycle of poplar plantations. Our research demonstrated that stand age significantly affected Rs, showing a decreasing trend in the Rs rate as stand age increased (Figure 1). The decrease trend is also reported in the *Populus balsamifera* L. and *Populus davidiana* Dode plantations in Xinjiang and Hebei in China, with the Rs rate of young forests being the highest, and the Rs significantly decreasing with the increase of stand age (Gong et al., 2012; Yan et al., 2011; Zhao et al., 2016). In contrast, there is no significant change in Rs with the increase of stand age in the hybrid poplar plantations (Saurette et al., 2008). The findings suggest that the effect of stand age on Rs can vary depending on the type of tree species and planting techniques, potentially attributed to disparities in the quality of the soil carbon pool and the amount of root biomass (Xiao et al., 2014).

In this study, stand age significantly influenced Rs by altering environmental conditions such as soil temperature and nutrients (Figure 6; Supplementary Figure S8). With the increase in stand age, the canopy structure gradually undergoes transformation. Mature forests typically possess denser canopies, reducing the amount of solar radiation reaching the ground surface and thereby modulating understory temperature and humidity (McCarthy and Brown, 2006). During warm seasons, the dense canopy mitigates direct sunlight on the soil surface, leading to lower ST. This reduced ST subsequently slows down microbial metabolic activities, resulting in decreased Rs rates. Conversely, in younger stands, the sparser canopy allows more sunlight to penetrate the soil surface, elevating ST and enhancing Rs. This study confirms this phenomenon, with significantly higher ST in 1a and 2a stands compared to older stands, and much higher Rs values in summer (June-August) than in spring (March-May) and autumn (September-November) (Figure 4). This aligns with the positive correlation between ST and Rs observed in previous studies (Wang et al., 2023). Additionally, while SM can potentially influence Rs under extreme conditions (Raich and Potter, 1995) no significant correlation between Rs and SM was detected in this study (Supplementary Figure S8). This may be attributed to the fact that during the growing season in the study area, SM in the poplar plantations was relatively abundant, posing no constraints on microbial and root respiration (Nottingham et al., 2020).

As stand age increases, the patterns of tree absorption and utilization of soil nutrients also undergo changes, subsequently influencing Rs. Young stands grow rapidly with high nutrient demands, potentially leading to a decrease in available nutrients such as nitrogen and available potassium in the soil, both of which are crucial elements for microbial respiration. The deficiency of these nutrients can inhibit microbial activity, thereby reducing Rs. In contrast, in mature stands, tree growth slows down, reducing nutrient demands, and soil nutrient content may stabilize, supporting more stable microbial activities (Rodríguez-Soalleiro et al., 2018). Concurrently, with the increase in stand age, the accumulation of plant residues and litter in the soil enhances the organic carbon content (Yang et al., 2014), providing a substrate source for microbial respiration. However, excessive organic carbon may also impact soil aeration, potentially inhibiting microbial activity.

Changes in Rs can also be explained by substrate carbon and nitrogen effectiveness and soil carbon fractions (SOC, MBC, DOC, EOC) (Tedeschi et al., 2006; Wu et al., 2020). Compared to soil recalcitrant carbon SOC, MBC, DOC and EOC are more readily available for direct use by soil microorganisms. In this study, soil carbon fractions (SOC, ECO, DOC/SOC, EOC/SOC), TN, NO_3_^−^-N, and Litter C/N (*p* < 0.001) were significantly correlated with Rs (Supplementary Figures S2, S4), and may be attributed to the strong response of Rs to substrate carbon and nitrogen effectiveness (Wang et al., 2017; Yang et al., 2022). Therefore, as stand age increases, changes in soil carbon-to-nitrogen ratios and litter carbon-to-nitrogen ratios both affect microbial biomass and activity, subsequently influencing Rs (Tu et al., 2013). In forest systems, inputs of litter increase SOC while decreasing soil nitrogen content, resulting in an increase in the soil C/N. Therefore, changes in both soil carbon and nitrogen, as well as litter carbon and nitrogen, affect soil microbial biomass and microbial activity, leading to changes in heterotrophic respiration, which is a component of total Rs (Tu et al., 2013). In this paper, the SOC decreased with stand age overall, it began to show a certain upward trend after 7a and 8a (Supplementary Figure S2). This may be attributed to the fact that poplars are designed to grow rapidly at younger ages, thereby absorbing large amounts of nutrient elements from the soil and accumulating biomass (Rodríguez-Soalleiro et al., 2018). After the stage of 7a and 8a, the plant growth rate slows down and nutrient uptake begins to decrease, allowing the retention and accumulation of apoplastic and root organic matter (Yang et al., 2014).

### 4.2 Changes of soil microbial communities with stand age

The results of our PERMANOVA analyses revealed that increased stand age significantly influenced the structure of soil bacterial and fungal communities (Supplementary Figure S7). Furthermore, our PLS-PM analyses attributed this effect to changes in soil properties brought about by increased stand age (Figure 6), which consequently altered the ecological strategies of microorganisms (Marques et al., 2014). Some studies have reported that increased root biomass stimulates secretions into the soil as a key factor contributing to increased soil microbial community diversity (Fraser et al., 2017), and if this is the case, we hypothesise that soil microbial diversity increased with stand age. However, our results showed that soil bacterial community diversity decreased with stand age, but soil fungal community diversity showed an increased trend (Supplementary Figure S6). This was possibly due to a decreased root secretion with age and the fact that the fungal community was more resistant to the effects of age than the bacterial community. Another explanation is that bacterial communities have a smaller ecological niche in the soil than fungal communities, have a smaller symbiotic relationship with plants than fungi, and provide less significant feedbacks to plants and soil than fungal communities (Sun et al., 2017). Thus fungal community diversity has a more positive direct or indirect effect on stand age. At the same time, stand age also altered the relative abundance of major bacterial phyla (Proteobacteria, Acidobacteriota, and Actinobacteriota) and fungal phyla (Ascomycota and Basidiomycota) (Figure 2). This result is similar to findings from poplar plantations in other regions (Wu et al., 2021). Proteobacteria and Actinobacteria were classified as copiotrophic bacteria, while Ascomycota was classified as copiotrophic fungi. Both copiotrophic bacteria and fungi belong to fast-growing taxa (r-strategists) and prefer nutrient-rich environments. On the other hand, Basidiomycota are classified as oligotrophic and belong to slow-growing taxa (K-strategists), which are better suited to grow in environments with lower nutrient concentrations. Meanwhile, the relative abundance of r-fungi (Ascomycota) exhibits an overall downward trend with the increase of stand age, while the relative abundance of K-fungi (Basidiomycota) displays an overall upward trend. This shift indicates that K-strategy fungi become more dominant as stand age increases.

Stand age, climate, soil properties and plant species were all important predictors of soil microbial community structure (Chen et al., 2021a; Marques et al., 2014). The structure of microbial communities is influenced by soil pH, permeability, and physicochemical properties (Krause et al., 2017; Lupwayi et al., 2017). This coincides with our finding that the diversity and structure of soil microbial communities varied significantly with stand age in relation to soil pH, and physico-chemical properties such as SBD, AK, NO_3_^−^-N, DOC/SOC, and litter C/N (Figure 3). Vitali et al. (2016) observed that soil microbial communities in poplar plantations are influenced by pH. Our results confirmed this finding and further supported the notion that soil microbial communities in poplar plantations of varying stand ages are also affected by pH. Additionally, Ascomycota and Basidiomycota may also influence the changes in soil pH and SBD (Zhao et al., 2018).

Prediction of ecological functions of soil bacterial and fungal communities using FAPROTAX and FUNGuild showed that soil microbial functional groups changed significantly with stand age (Supplementary Figure S5). Among the bacterial functional groups, the abundance of nitrate reduction and photoautotrophy generally decreases with increasing stand age, while the abundance of nitrogen fixation generally increases with increasing stand age (Supplementary Figure S5A). However, Yan et al. (2020) study of secondary succession in *Quercus liaotungensis* forests found that the relative abundance of microbial functional groups associated with soil carbon and nitrogen cycling increased with succession, which should be attributed to the low levels of both SOC and TN in our study area. As the understory vegetation ecosystem recovers as the stand ages, the relative abundance of nitrogen fixation bacteria increases (Blaud et al., 2018). Only dung saprotroph and litter saprotroph among the saprotrophic types showed a significant decrease with stand age (Supplementary Figure S5B). Most saprophytic bacteria are in the Ascomycetes phylum and are important decomposers of soil as they are able to break down the complex structure of organic matter in the soil (Paungfoo-Lonhienne et al., 2015). Therefore, with increasing stand age, the saprotrophic functional groups may be influenced by changes in the relative abundance of the Ascomycota phylum.

When discussing changes in soil microbial functional groups in poplar plantations with increasing stand age, we relied solely on two databases for prediction. This limitation may restrict the comprehensiveness of our conclusions, as it may not fully capture the diversity and dynamic changes of soil microbial functional groups. In order to better understand how stand age affects changes in soil microbial function, future studies should expand samples and use better sequencing methods.

### 4.3 Important factors affecting Rs

Based on the regression analysis model and Pearson’s correlation analysis, we observed that changes in Rs depend strongly on the microbial properties at the community level (Figure 5; Supplementary Figure S8). In addition to soil properties, changes in temperature often affect the structure of soil microbial communities. For instance, fungal communities prefer cooler environments relative to bacterial communities. According to the Carbon-Quality-Temperature hypothesis, recalcitrant carbon decomposition with higher activation energy possesses higher temperature sensitivity compared to the decomposition of labile carbon (Fierer et al., 2005; Wang et al., 2018). And soil microbes are more active in carbon-rich conditions. Therefore, the reasons for the changes in Rs with stand age can be explained by the decreasing trends in soil carbon and nitrogen content and temperature with stand age. This viewpoint is well-supported by ample evidence, revealing the close relationships and interactions among stand age, soil properties, ST and bacterial community structure.

Specific microbial communities and ecological clusters are important factors in microbial prediction of Rs (Wang et al., 2021). In conclusion, microorganisms associated with carbon mineralisation (Actinobacteriota and Ascomycota) and microbial r-/K strategy ratio (fungal copiotroph/oligotroph ratio) were the main biological factors that predicted a decline in soil respiration with age in poplar plantations. Copiotrophic and oligotrophic have different ecological functions in using carbon for respiration due to different substrate utilisation strategies. R-strategy communities prefer nutrient-rich and warm environments over K-strategy communities and use the majority of the acquired energy for their own growth and reproduction, thus reducing respiration efficiency while having more efficient carbon utilisation. In contrast, K-strategy tends to preferentially utilise recalcitrant carbon, which requires a large amount of energy consumption, and so devotes the majority of its energy and resources to respiration (Liu et al., 2020b; Malik et al., 2020). The positive correlation of Ascomycota and Fungal C/O with Rs further confirmed that Rs decreased with the distribution of soil microbial r-strategies under increasing stand age.

We observed microbial r-/K strategy ratio (fungal copiotroph/oligotroph ratio), microbial community diversity (e.g., Shannon and NMDS1 for bacteria and fungi), and microbial communities associated with carbon mineralisation (Actinobacteriota and Ascomycota) as important biotic factors affecting Rs. However, soil microbial communities are often influenced by a number of other abiotic factors such as soil physico-chemical properties. Thus, such abiotic factors are also important drivers of Rs. For example, our data indicates that SOC and NO_3_^−^-N are abiotic factors that affect soil carbon flux, consistent with the findings reported for Rs in *Pinus massoniana* plantations (Yu et al., 2019). In general, a high soil C/N ratio inhibits microbial decomposition (He et al., 2018). However, although no significant effect of soil microorganisms on Rs was observed in PLS-PM analysis, the importance of soil microorganisms was verified by both the regression analysis model and the correlation analysis. Thus, our findings emphasised the potential importance of soil microbial communities and ecological clusters in predicting Rs.

In general, the Rs rate in poplar plantations decreases with increasing stand age, as stand age influences environmental conditions such as soil temperature (ST), nutrients, and microbial communities. This suggests that with increasing stand age, the carbon sink capacity of poplar plantations may be enhanced, since lower Rs corresponds to reduced soil carbon loss. Thus, older poplar plantations may be more effective in carbon sequestration than younger ones, contributing to global carbon storage. In the context of global warming, Rs typically increases with rising temperatures, leading to higher CO₂ emissions. However, our study shows that as stand age increases, Rs decline, suggesting that older forests may act as crucial carbon sinks, mitigating climate change. A lower Rs rate may offset the adverse effects of climate change by delaying soil carbon release. Therefore, we propose extending the growth cycles of poplar plantations, particularly maintaining older stands, to aid in carbon sequestration and climate mitigation efforts. In forest management and land-use planning, focusing on preserving older forests could optimize carbon sequestration. Additionally, poplars are significant sources of volatile organic compound (VOC) emissions, which may result in carbon loss and are linked to stand age. Future research should focus on stand age’s effect on the ecological carbon dynamics of poplar plantations, which is crucial for assessing ecological services across different ages.

## 5 Conclusion

Rs decreased with increasing stand age in poplar plantations throughout the growth cycle. The microbial r-strategies were the key biotic factors that influenced Rs in different-age poplar plantations. Other abiotic factors such as ST, pH, SOC and NO_3_^−^-N, and litter C/N were also important drivers of Rs. Soil properties such as pH and SBD also significantly affected soil microbial community diversity and composition, and altered the ecological strategies of microbial communities, which in turn impacted altered Rs. Our study provides new insights into understanding the changes in Rs in poplar plantations in Northeast China influenced by stand age. Meanwhile, the study highlighted the potential link between the combination of different ecological taxa of soil microbes and Rs, which is of great significance for the prediction of soil carbon dynamics in poplar plantation ecosystems in the context of global warming.

## Data Availability

The bacterial and fungal DNA sequences of the soil samples have been deposited in the SRA (Sequence Read Archive) of the NCBI database, with the accession number PRJNA1180506.

## References

[ref1] Bastida F. Lopez-Mondejar R. Baldrian P. Andres-Abellan M. Jehmlich N. Torres I. F.. (2019). When drought meets forest management: effects on the soil microbial community of a Holm oak forest ecosystem. Sci. Total Environ. 662, 276–286. doi: 10.1016/j.scitotenv.2019.01.233, PMID: 30690362

[ref2] Blaud A. van der Zaan B. Menon M. Lair G. J. Zhang D. Y. Huber P.. (2018). The abundance of nitrogen cycle genes and potential greenhouse gas fluxes depends on land use type and little on soil aggregate size. Appl. Soil Ecol. 125, 1–11. doi: 10.1016/j.apsoil.2017.11.026

[ref3] Bonan G. B. (2008). Forests and climate change: forcings, feedbacks, and the climate benefits of forests. Science 320, 1444–1449. doi: 10.1126/science.1155121, PMID: 18556546

[ref4] Bond-Lamberty B. Thomson A. (2010). Temperature-associated increases in the global soil respiration record. Nature 464, 579–582. doi: 10.1038/nature08930 20336143

[ref5] Castro S. P. Cleland E. E. Wagner R. Al Sawad R. Lipson D. A. (2019). Soil microbial responses to drought and exotic plants shift carbon metabolism. ISME J. 13, 1776–1787. doi: 10.1038/s41396-019-0389-9, PMID: 30872806 PMC6776022

[ref6] Chen L. F. He Z. B. Wu X. R. Du J. Zhu X. Lin P. F.. (2021a). Linkages between soil respiration and microbial communities following afforestation of alpine grasslands in the northeastern Tibetan plateau. Appl. Soil Ecol. 161:103882. doi: 10.1016/j.apsoil.2021.103882

[ref7] Chen L. F. He Z. B. Zhao W. Z. Kong J. Q. Gao Y. J. (2021b). Empirical evidence for microbial regulation of soil respiration in alpine forests. Ecol. Indic. 126:107710. doi: 10.1016/j.ecolind.2021.107710

[ref8] Dixon R. K. Solomon A. M. Brown S. Houghton R. A. Trexier M. C. Wisniewski J. (1994). Carbon pools and flux of global forest ecosystems. Science 263, 185–190. doi: 10.1126/science.263.5144.185, PMID: 17839174

[ref9] Fang J. Y. Yu G. R. Liu L. L. Hu S. J. Chapin F. S. III (2018). Climate change, human impacts, and carbon sequestration in China. Proc. Natl. Acad. Sci. U.S.A. 115, 4015–4020. doi: 10.1073/pnas.1700304115, PMID: 29666313 PMC5910806

[ref10] Feng J. G. Wang J. S. Song Y. J. Zhu B. (2018). Patterns of soil respiration and its temperature sensitivity in grassland ecosystems across China. Biogeosciences 15, 5329–5341. doi: 10.5194/bg-15-5329-2018

[ref11] Fierer N. Bradford M. A. Jackson R. B. (2007). Toward an ecological classification of soil bacteria. Ecology 88, 1354–1364. doi: 10.1890/05-1839 17601128

[ref12] Fierer N. Craine J. M. McLauchlan K. Schimel J. P. (2005). Litter quality and the temperature sensitivity of decomposition. Ecology 86, 320–326. doi: 10.1890/04-1254

[ref13] Fraser T. D. Lynch D. H. Gaiero J. Khosla K. Dunfield K. E. (2017). Quantification of bacterial non-specific acid (*phoC*) and alkaline (*phoD*) phosphatase genes in bulk and rhizosphere soil from organically managed soybean fields. Appl. Soil Ecol. 111, 48–56. doi: 10.1016/j.apsoil.2016.11.013

[ref14] Gielen B. Ceulemans R. (2001). The likely impact of rising atmospheric CO_2_ on natural and managed *Populus*: a literature review. Environ. Pollut. 115, 335–358. doi: 10.1016/S0269-7491(01)00226-3, PMID: 11789917

[ref15] Gong J. R. Ge Z. W. An R. Duan Q. W. You X. Huang Y. M. (2012). Soil respiration in poplar plantations in northern China at different forest ages. Plant Soil 360, 109–122. doi: 10.1007/s11104-011-1121-3

[ref16] Han S. Wang A. (2023). Belowground bacterial communities and carbon components contribute to soil respiration in a subtropical forest. Plant Soil 501, 125–137. doi: 10.1007/s11104-023-06257-3

[ref17] He Z. B. Chen L. F. Du J. Zhu X. Lin P. F. Li J.. (2018). Responses of soil organic carbon, soil respiration, and associated soil properties to long-term thinning in a semiarid spruce plantation in northwestern China. Land Degrad. Dev. 29, 4387–4396. doi: 10.1002/ldr.3196

[ref18] Huang N. Wang L. Song X. P. Black T. A. Jassal R. S. Myneni R. B.. (2020). Spatial and temporal variations in global soil respiration and their relationships with climate and land cover. Sci. Adv. 6:eabb8508. doi: 10.1126/sciadv.abb8508, PMID: 33028522 PMC7541079

[ref19] Krause S. M. B. Johnson T. Karunaratne Y. S. Fu Y. F. Beck D. A. C. Chistoserdova L.. (2017). Lanthanide-dependent cross-feeding of methane-derived carbon is linked by microbial community interactions. Proc. Natl. Acad. Sci. U.S.A. 114, 358–363. doi: 10.1073/pnas.1619871114, PMID: 28028242 PMC5240692

[ref20] Lefroy R. D. B. Blair G. J. Strong W. M. (1993). Changes in soil organic matter with cropping as measured by organic carbon fractions and ^13^C natural isotope abundance. Plant Soil 155-156, 399–402. doi: 10.1007/BF00025067

[ref21] Li H. Yang S. Semenov M. V. Yao F. Ye J. Bu R. C.. (2021). Temperature sensitivity of SOM decomposition is linked with a K-selected microbial community. Glob. Change Biol. 27, 2763–2779. doi: 10.1111/gcb.15593, PMID: 33709545

[ref22] Liu Y. R. Delgado-Baquerizo M. Wang J. T. Hu H. W. Yang Z. M. He J. Z. (2018a). New insights into the role of microbial community composition in driving soil respiration rates. Soil Biol. Biochem. 118, 35–41. doi: 10.1016/j.soilbio.2017.12.003

[ref23] Liu Y. R. Delgado-Baquerizo M. Yang Z. M. Feng J. Zhu J. Huang Q. Y. (2020b). Microbial taxonomic and functional attributes consistently predict soil CO_2_ emissions across contrasting croplands. Sci. Total Environ. 702:134885. doi: 10.1016/j.scitotenv.2019.134885, PMID: 31731121

[ref24] Liu S. E. Wang H. Tian P. Yao X. Sun H. Wang Q. K.. (2020). Decoupled diversity patterns in bacteria and fungi across continental forest ecosystems. Soil Biol. Biochem. 144:107763. doi: 10.1016/j.soilbio.2020.107763

[ref25] Liu M. H. Wei Y. Q. Lian L. Wei B. Bi Y. X. Liu N.. (2023). Macrofungi promote SOC decomposition and weaken sequestration by modulating soil microbial function in temperate steppe. Sci. Total Environ. 899:165556. doi: 10.1016/j.scitotenv.2023.165556, PMID: 37459997

[ref26] Liu X. P. Zhang W. J. Cao J. S. Shen H. T. Zeng X. H. Yu Z. Q.. (2013). Carbon storages in plantation ecosystems in sand source areas of North Beijing, China. Plos One. 8:11. doi: 10.1371/journal.pone.0082208 PMC3861385 24349223

[ref27] Louca S. Parfrey L. W. Doebeli M. (2016). Decoupling function and taxonomy in the global ocean microbiome. Science 353, 1272–1277. doi: 10.1126/science.aaf4507, PMID: 27634532

[ref28] Lu F. Hu H. F. Sun W. J. Zhu J. J. Liu G. B. Zhou W. M.. (2018). Effects of national ecological restoration projects on carbon sequestration in China from 2001 to 2010. Proc. Natl. Acad. Sci. U.S.A. 115, 4039–4044. doi: 10.1073/pnas.1700294115, PMID: 29666317 PMC5910802

[ref29] Lupwayi N. Z. Larney F. J. Blackshaw R. E. Kanashiro D. A. Pearson D. C. (2017). Phospholipid fatty acid biomarkers show positive soil microbial community responses to conservation soil management of irrigated crop rotations. Soil Tillage Res. 168, 1–10. doi: 10.1016/j.still.2016.12.003

[ref30] Ma X. L. Jiang S. J. Zhang Z. Q. Wang H. Song C. He J. S. (2023). Long-term collar deployment leads to bias in soil respiration measurements. Methods Ecol. Evol. 14, 981–990. doi: 10.1111/2041-210x.14056

[ref31] Ma Y. C. Piao S. L. Sun Z. Z. Lin X. Wang T. Yue C.. (2014). Stand ages regulate the response of soil respiration to temperature in a *Larix principis-rupprechtii* plantation. Agric. For. Meteorol. 184, 179–187. doi: 10.1016/j.agrformet.2013.10.008

[ref32] Malik A. A. Martiny J. B. H. Brodie E. L. Martiny A. C. Treseder K. K. Allison S. D. (2020). Defining trait-based microbial strategies with consequences for soil carbon cycling under climate change. ISME J. 14, 1–9. doi: 10.1038/s41396-019-0510-0, PMID: 31554911 PMC6908601

[ref33] Marques J. M. da Silva T. F. Vollu R. E. Blank A. F. Ding G. C. Seldin L.. (2014). Plant age and genotype affect the bacterial community composition in the tuber rhizosphere of field-grown sweet potato plants. FEMS Microbiol. Ecol. 88, 424–435. doi: 10.1111/1574-6941.12313, PMID: 24597529

[ref34] Masson-Delmotte V. Zhai P. Pirani S. Connors C. Pean S. Berger N.. (2021). “IPCC, 2021: summary for policymakers” in The physical science basis. Contribution of Working Group I to the Sixth Assessment Report of the Intergovernmental Panel on Climate Change (Cambridge: Cambridge University Press).

[ref35] McCarthy D. R. Brown K. J. (2006). Soil respiration responses to topography, canopy cover, and prescribed burning in an oak-hickory forest in southeastern Ohio. For. Ecol. Manag. 237, 94–102. doi: 10.1016/j.foreco.2006.09.030

[ref36] National Forestry and Grassland Administration. (2019). China forest resources assessment. China Forest Resources Report: 2014-2018. China Forestry Publishing House.

[ref37] Nguyen N. H. Song Z. W. Bates S. T. Branco S. Tedersoo L. Menke J.. (2016). FUNGuild: an open annotation tool for parsing fungal community datasets by ecological guild. Fungal Ecol. 20, 241–248. doi: 10.1016/j.funeco.2015.06.006

[ref38] Nottingham A. T. Meir P. Velasquez E. Turner B. L. (2020). Soil carbon loss by experimental warming in a tropical forest. Nature 584, 234–237. doi: 10.1038/s41586-020-2566-4, PMID: 32788738

[ref39] Nottingham A. T. Scott J. J. Saltonstall K. Broders K. Montero-Sanchez M. Puspok J.. (2022). Microbial diversity declines in warmed tropical soil and respiration rise exceed predictions as communities adapt. Nat. Microbiol. 7, 1650–1660. doi: 10.1038/s41564-022-01200-1, PMID: 36065063

[ref40] Pan Y. D. Birdsey R. A. Fang J. Y. Houghton R. Kauppi P. E. Kurz W. A.. (2011). A large and persistent carbon sink in the world’s forests. Science 333, 988–993. doi: 10.1126/science.1201609, PMID: 21764754

[ref41] Paungfoo-Lonhienne C. Yeoh Y. K. Kasinadhuni N. R. P. Lonhienne T. G. A. Robinson N. Hugenholtz P.. (2015). Nitrogen fertilizer dose alters fungal communities in sugarcane soil and rhizosphere. Sci. Rep. 5:8678. doi: 10.1038/srep08678, PMID: 25728892 PMC5155403

[ref42] Pilipovic A. Headlee W. L. Zalesny R. S. Jr. Pekec S. Bauer E. O. (2022). Water use efficiency of poplars grown for biomass production in the Midwestern United States. Glob. Change Biol. Bioenergy. 14, 287–306. doi: 10.1111/gcbb.12887

[ref43] Powers M. Kolka R. Bradford J. Palik B. Jurgensen M. (2018). Forest floor and mineral soil respiration rates in a northern Minnesota red pine chronosequence. Forests 9:15. doi: 10.3390/f9010016

[ref44] Raich J. W. Potter C. S. (1995). Global patterns of carbon dioxide emissions from soils. Glob. Biogeochem. Cycles 9, 23–36. doi: 10.1029/94GB02723

[ref45] Reay D. Sabine C. Smith P. Hymus G. (2007). Spring-time for sinks. Nature 446, 727–728. doi: 10.1038/446727a, PMID: 17429376

[ref46] Rodríguez-Soalleiro R. Eimil-Fraga C. Gomez-Garcia E. Garcia-Villabrille J. D. Rojo-Alboreca A. Munoz F.. (2018). Exploring the factors affecting carbon and nutrient concentrations in tree biomass components in natural forests, forest plantations and short rotation forestry. For. Ecosyst. 5:35. doi: 10.1186/s40663-018-0154-y

[ref47] Sanchez G. Trinchera L. Sanchez M. G. FactoMineR S. (2013). Package ‘plspm’. State College, PA: Citeseer.

[ref48] Saurette D. D. Chang S. X. Thomas B. R. (2008). Autotrophic and heterotrophic respiration rates across a chronosequence of hybrid poplar plantationsin northern Alberta. Can. J. Soil Sci. 88, 261–272. doi: 10.4141/CJSS07005

[ref49] Siles J. A. Margesin R. (2016). Abundance and diversity of bacterial, archaeal, and fungal communities along an altitudinal gradient in alpine forest soils: what are the driving factors? Microb. Ecol. 72, 207–220. doi: 10.1007/s00248-016-0748-2, PMID: 26961712 PMC4902835

[ref50] Sun S. Li S. Avera B. N. Strahm B. D. Badgley B. D. (2017). Soil bacterial and fungal communities show distinct recovery patterns during forest ecosystem restoration. Appl. Environ. Microbiol. 83:e00966. doi: 10.1128/AEM.00966-17, PMID: 28476769 PMC5494632

[ref51] Tang J. W. Bolstad P. V. Martin J. G. (2009). Soil carbon fluxes and stocks in a Great Lakes forest chronosequence. Glob. Change Biol. 15, 145–155. doi: 10.1111/j.1365-2486.2008.01741.x

[ref52] Tedeschi V. Rey A. Manca G. Valentini R. Jarvis P. G. Borghetti M.. (2006). Soil respiration in a Mediterranean oak forest at different developmental stages after coppicing. Glob. Change Biol. 12, 110–121. doi: 10.1111/j.1365-2486.2005.01081.x

[ref7001] Teuber M. Zimmer I. Kreuzwiese J. Ache P. Polle A. Rennenberg H.. (2007). VOC emissions of Grey poplar leaves as affected by salt stress and different N sources. Plant Biology 10, 86–96. doi: 10.1111/j.1438-8677.2007.00015.x 18211549

[ref53] Tu L. H. Hu T. X. Zhang J. Li X. W. Hu H. L. Liu L.. (2013). Nitrogen addition stimulates different components of soil respiration in a subtropical bamboo ecosystem. Soil Biol. Biochem. 58, 255–264. doi: 10.1016/j.soilbio.2012.12.005

[ref54] Vitali F. Mastromei G. Senatore G. Caroppo C. Casalone E. (2016). Long lasting effects of the conversion from natural forest to poplar plantation on soil microbial communities. Microbiol. Res. 182, 89–98. doi: 10.1016/j.micres.2015.10.002 26686617

[ref55] Wang H. H. Huang W. D. He Y. Z. Zhu Y. Z. (2023). Effects of warming and precipitation reduction on soil respiration in Horqin sandy grassland, northern China. Catena 233:107470. doi: 10.1016/j.Catena.2023.107470

[ref57] Wang R. Sun Q. Q. Wang Y. Liu Q. F. Du L. L. Zhao M.. (2017). Temperature sensitivity of soil respiration: synthetic effects of nitrogen and phosphorus fertilization on Chinese Loess Plateau. Sci. Total Environ. 574, 1665–1673. doi: 10.1016/j.scitotenv.2016.09.001, PMID: 27614858

[ref58] Wang Q. Sun Q. W. Wang W. Z. Liu X. R. Song L. G. Hou L. Y. (2022). Effects of different native plants on soil remediation and microbial diversity in Jiulong Iron Tailings Area, Jiangxi. Forests 13:1106. doi: 10.3390/f13071106

[ref59] Wang C. Q. Xue L. Dong Y. H. Wei Y. H. Jiao R. J. (2018). Unravelling the functional diversity of the soil microbial community of Chinese fir plantations of different densities. Forests 9:532. doi: 10.3390/f9090532

[ref60] Wang X. X. Zhang W. Liu Y. Jia Z. J. Li H. Yang Y. F.. (2021). Identification of microbial strategies for labile substrate utilization at phylogenetic classification using a microcosm approach. Soil Biol. Biochem. 153:107970. doi: 10.1016/j.soilbio.2020.107970

[ref61] Wiseman P. E. Seiler J. R. (2004). Soil CO_2_ efflux across four age classes of plantation loblolly pine *Pinus taeda* L. on the Virginia Piedmont. For. Ecol. Manag. 192, 297–311. doi: 10.1016/j.foreco.2004.01.017

[ref62] Wu N. Li Z. Meng S. Wu F. (2021). Soil properties and microbial community in the rhizosphere of *Populus alba var. pyramidalis* along a chronosequence. Microbiol. Res. 250:126812. doi: 10.1016/j.micres.2021.126812, PMID: 34246038

[ref63] Wu X. Xu H. Tuo D. F. Wang C. Fu B. J. Lv Y. H.. (2020). Land use change and stand age regulate soil respiration by influencing soil substrate supply and microbial community. Geoderma 359:113991. doi: 10.1016/j.geoderma.2019.113991

[ref64] Xiao W. F. Ge X. G. Zeng L. X. Huang Z. L. Lei J. P. Zhou B. Z.. (2014). Rates of litter decomposition and soil respiration in relation to soil temperature and water in different-aged *Pinus massoniana* forests in the three gorges reservoir area, China. Plos One. 9:e101890. doi: 10.1371/journal.pone.0101890, PMID: 25004164 PMC4087021

[ref65] Yan B. S. Sun L. P. Li J. J. Liang C. Q. Wei F. R. Xue S.. (2020). Change in composition and potential functional genes of soil bacterial and fungal communities with secondary succession in *Quercus liaotungensis* forests of the Loess Plateau, western China. Geoderma 364:114199. doi: 10.1016/j.geoderma.2020.114199

[ref66] Yan M. Zhang X. Zhou G. Gong J. You X. (2011). Temporal and spatial variation in soil respiration of poplar plantations at different developmental stages in Xinjiang, China. J. Arid Environ. 75, 51–57. doi: 10.1016/j.jaridenv.2010.09.005

[ref67] Yang Q. Lei A. P. Li F. L. Liu L. N. Zan Q. J. Shin P. K. S.. (2014). Structure and function of soil microbial community in artificially planted *Sonneratia apetala* and *S. Caseolaris* forests at different stand ages in Shenzhen Bay, China. Mar. Pollut. Bull. 85, 754–763. doi: 10.1016/j.marpolbul.2014.02.024, PMID: 24629377

[ref68] Yang S. Wu H. Wang Z. R. Semenov M. V. Ye J. Yin L. M.. (2022). Linkages between the temperature sensitivity of soil respiration and microbial life strategy are dependent on sampling season. Soil Biol. Biochem. 172:108758. doi: 10.1016/j.soilbio.2022.108758

[ref69] Yu K. Y. Yao X. Deng Y. B. Lai Z. J. Lin L. C. Liu J. (2019). Effects of stand age on soil respiration in *Pinus massoniana* plantations in the hilly red soil region of southern China. Catena 178, 313–321. doi: 10.1016/j.catena.2019.03.038

[ref70] Zeng X. M. Feng J. Chen J. Delgado-Baquerizo M. Zhang Q. G. Zhou X. Q.. (2022). Microbial assemblies associated with temperature sensitivity of soil respiration along an altitudinal gradient. Sci. Total Environ. 820:153257. doi: 10.1016/j.scitotenv.2022.153257, PMID: 35065115

[ref71] Zhang F. G. Zhang Q. G. (2016). Microbial diversity limits soil heterotrophic respiration and mitigates the respiration response to moisture increase. Soil Biol. Biochem. 98, 180–185. doi: 10.1016/j.soilbio.2016.04.017

[ref72] Zhao X. Li F. D. Zhang W. J. Ai Z. P. Shen H. T. Liu X. P.. (2016). Soil respiration at different stand ages (5, 10, and 20/30 years) in coniferous (*Pinus tabulaeformis* Carriere) and deciduous (*Populus davidiana* Dode) plantations in a sandstorm source area. Forests 7:15. doi: 10.3390/f7080153

[ref73] Zhao F. Z. Ren C. J. Zhang L. Han X. H. Yang G. H. Wang J. (2018). Changes in soil microbial community are linked to soil carbon fractions after afforestation. Eur. J. Soil Sci. 69, 370–379. doi: 10.1111/ejss.12525

