# Supplementary material for: Effects of stand age and soil microbial communities on soil respiration throughout the growth cycle of poplar plantations in northeastern China
Source: Front Microbiol. 2024 Nov 27;15:1477571. doi: 10.3389/fmicb.2024.1477571 (PMC11631584; doi:10.3389/fmicb.2024.1477571)
Supplement: Supplementary file 1 [file Data_Sheet_1.pdf]

Supplementary Information

Effects of stand age on soil respiration and soil microbial communities throughout the growth cycle of poplar plantations in northeastern China

Xiangrong Liu [a,b,1], Lingyu Hou [a,b,1] , Changjun Ding [a,b,*], Xiaohua Su [a,b], Weixi Zhang [a,b], Zhongyi Pang [c], Yanlin Zhang [a,b], Qiwu Sun[a,b,*]

***Correspondence authors.**

E-mail addresses: changjund@126.com (C. Ding), sqw@caf.ac.cn (Q. Sun).

Research Institute of Forestry, Chinese Academy of Forestry, Beijing 100091, China

[1]These authors contributed equally to this work.

This file includes:

FIGURES S1 to S7

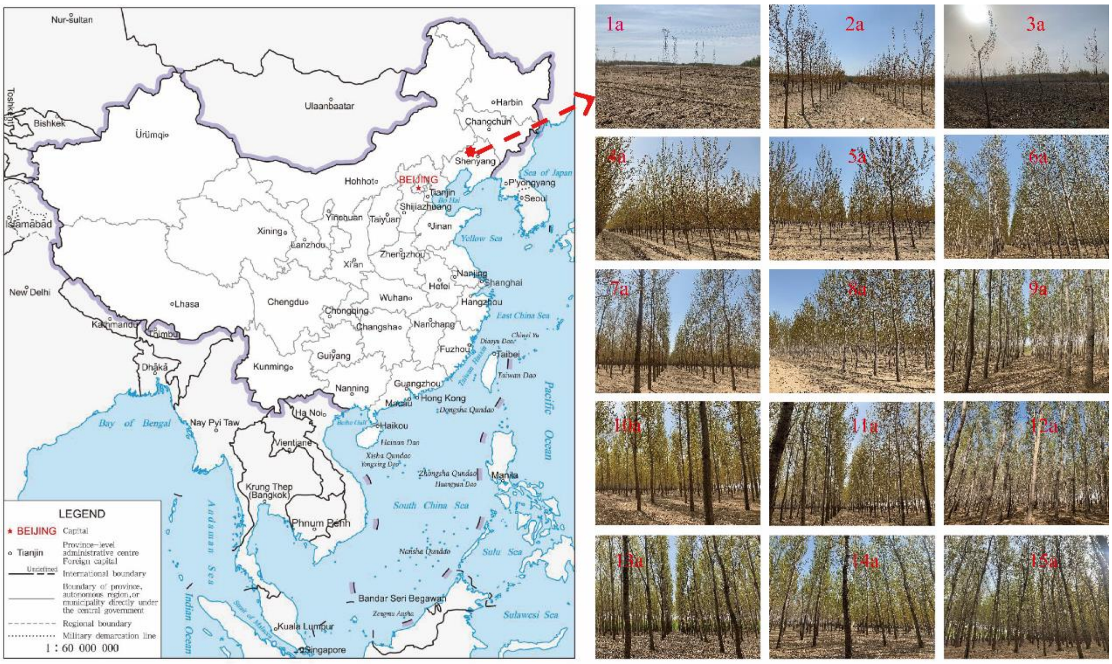

FIGURE S1. Location of sampling sites and photographs of various stand age stages.

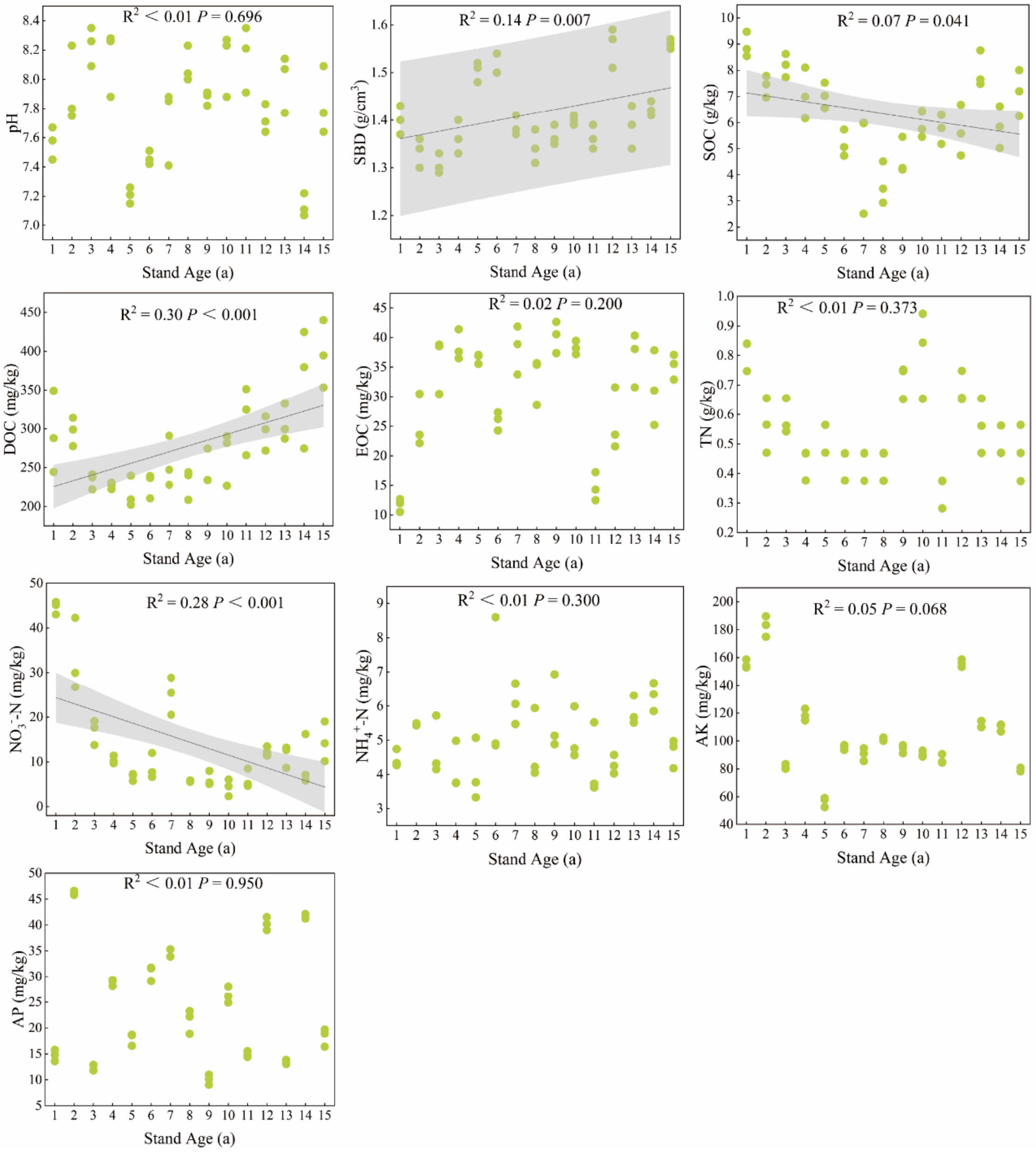

FIGURE S2. Relationship between soil physico-chemical properties and stand age. The solid black line indicates a significant linear relationship consistent with the regression model, and the shaded area indicates the fitted 95% confidence interval. SBD, soil bulk density; SOC, soil organic carbon; DOC, dissolved organic carbon; EOC, Easily oxidised organic carbon; TN, total nitrogen; NO_3^- -N, nitrate nitrogen; NH_4^+ -N, ammonium nitrogen; AK, available potassium; AP, Available phosphorus.

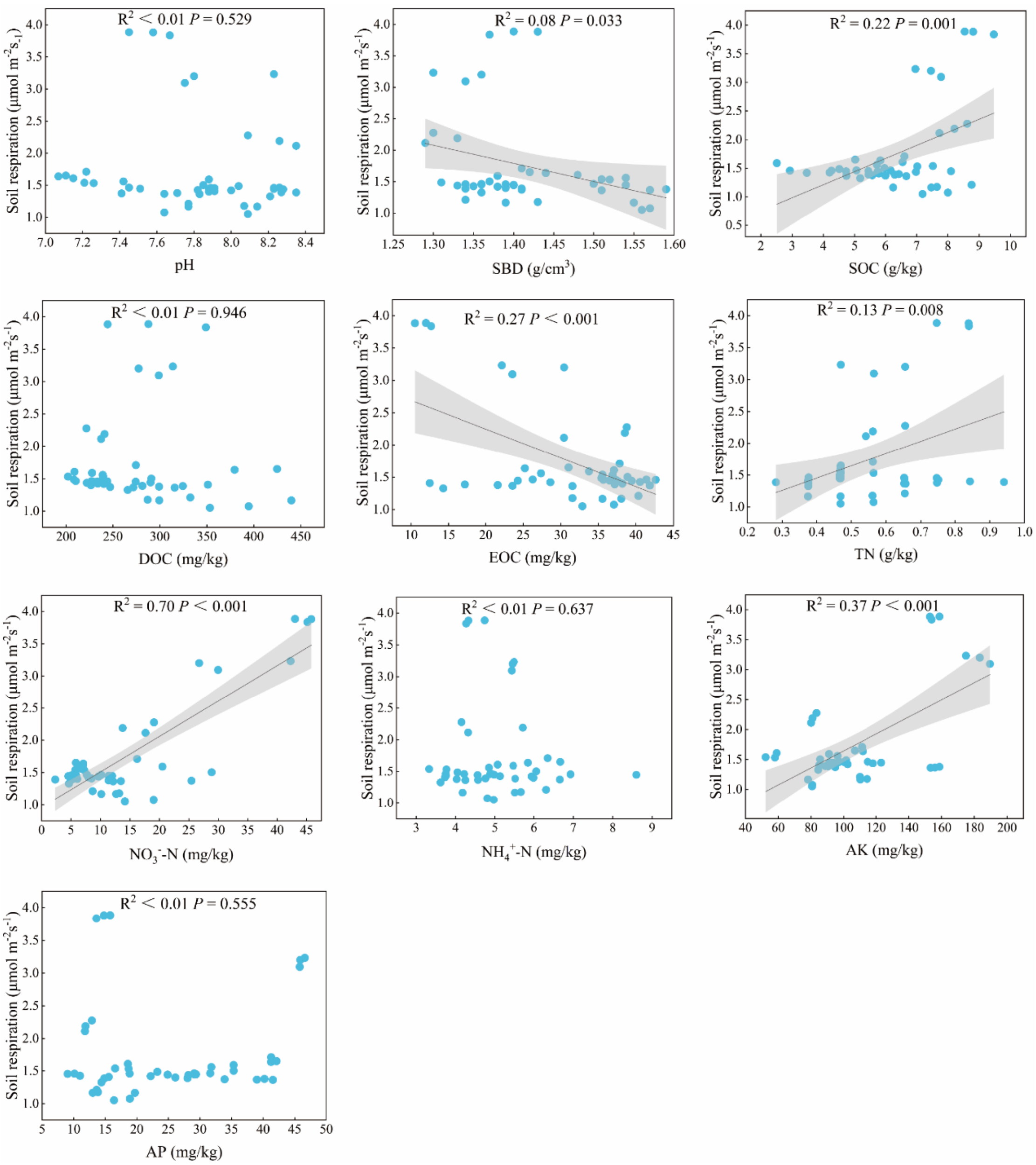

FIGURE S3. Relationship between soil physico-chemical properties and soil respiration. The solid black line indicates a significant linear relationship consistent with the regression model, and the shaded area indicates the fitted 95% confidence interval. SBD, soil bulk density; SOC, soil organic carbon; DOC, dissolved organic carbon; EOC, Easily oxidised organic carbon; TN, total nitrogen; NO_3^--N, nitrate nitrogen; NH_4^+-N, ammonium nitrogen; AK, available potassium; AP, Available phosphorus.

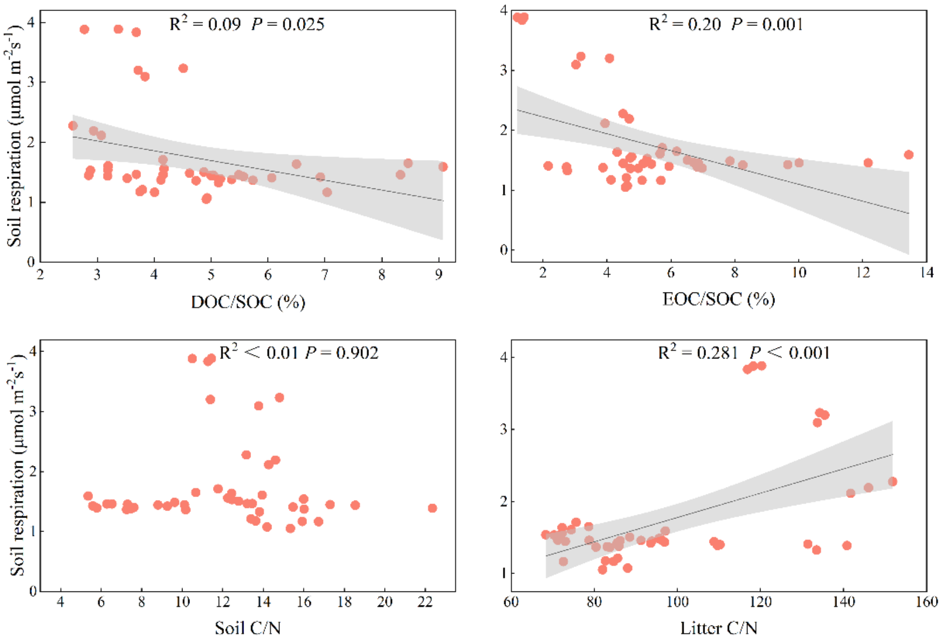

FIGURE S4. Relationship between soil respiration and DOC/SOC; EOC/SOC; soil C/N; plant litter C/N. The solid black line indicates a significant linear relationship consistent with the regression model, and the shaded area indicates the fitted 95% confidence interval.

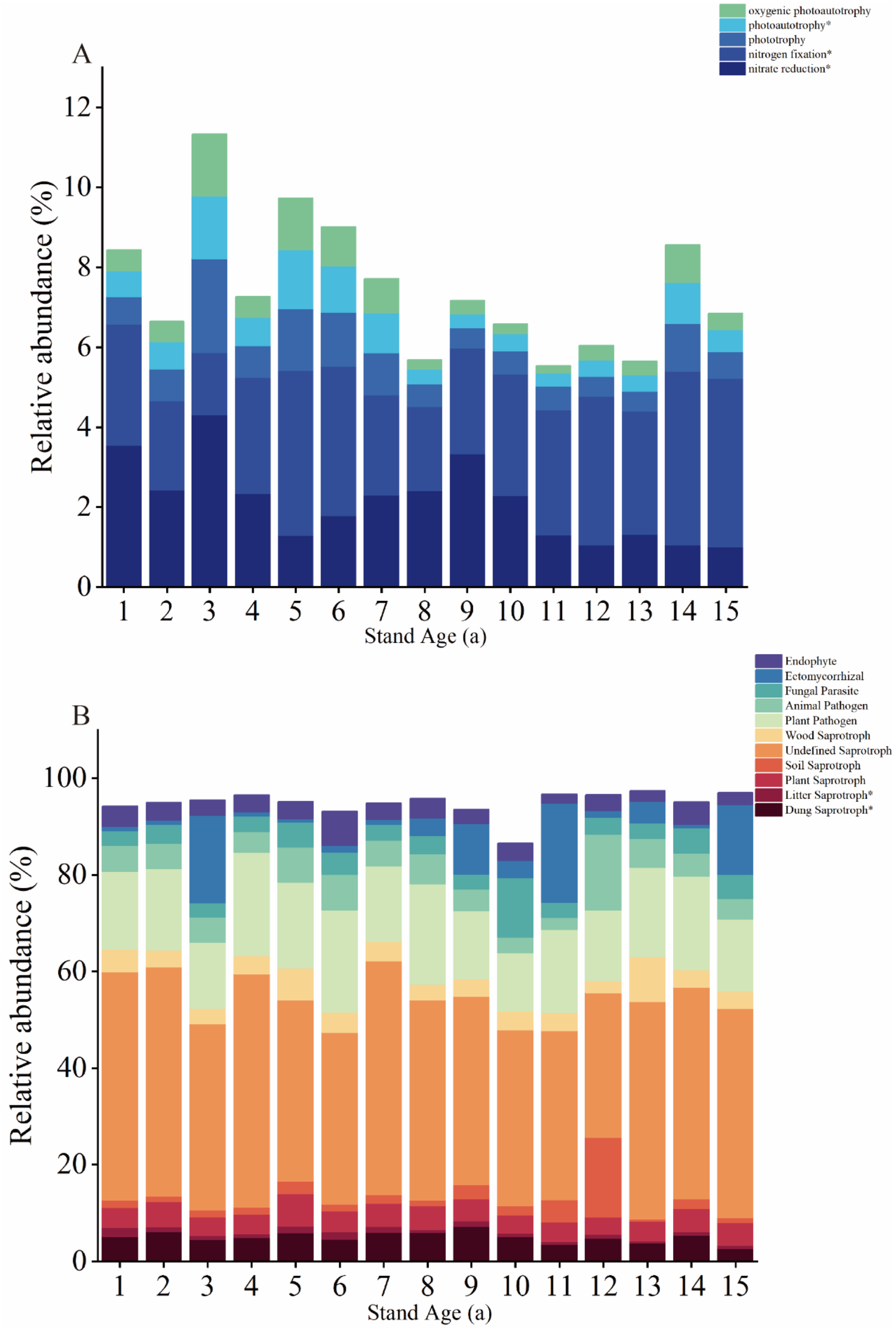

FIGURE S5. Relative abundance of bacterial (A) and fungal (B) functional guilds based on FAPROTAX and FUNGuild. * Significant differences ($P < 0.05$) were found between different stand ages.

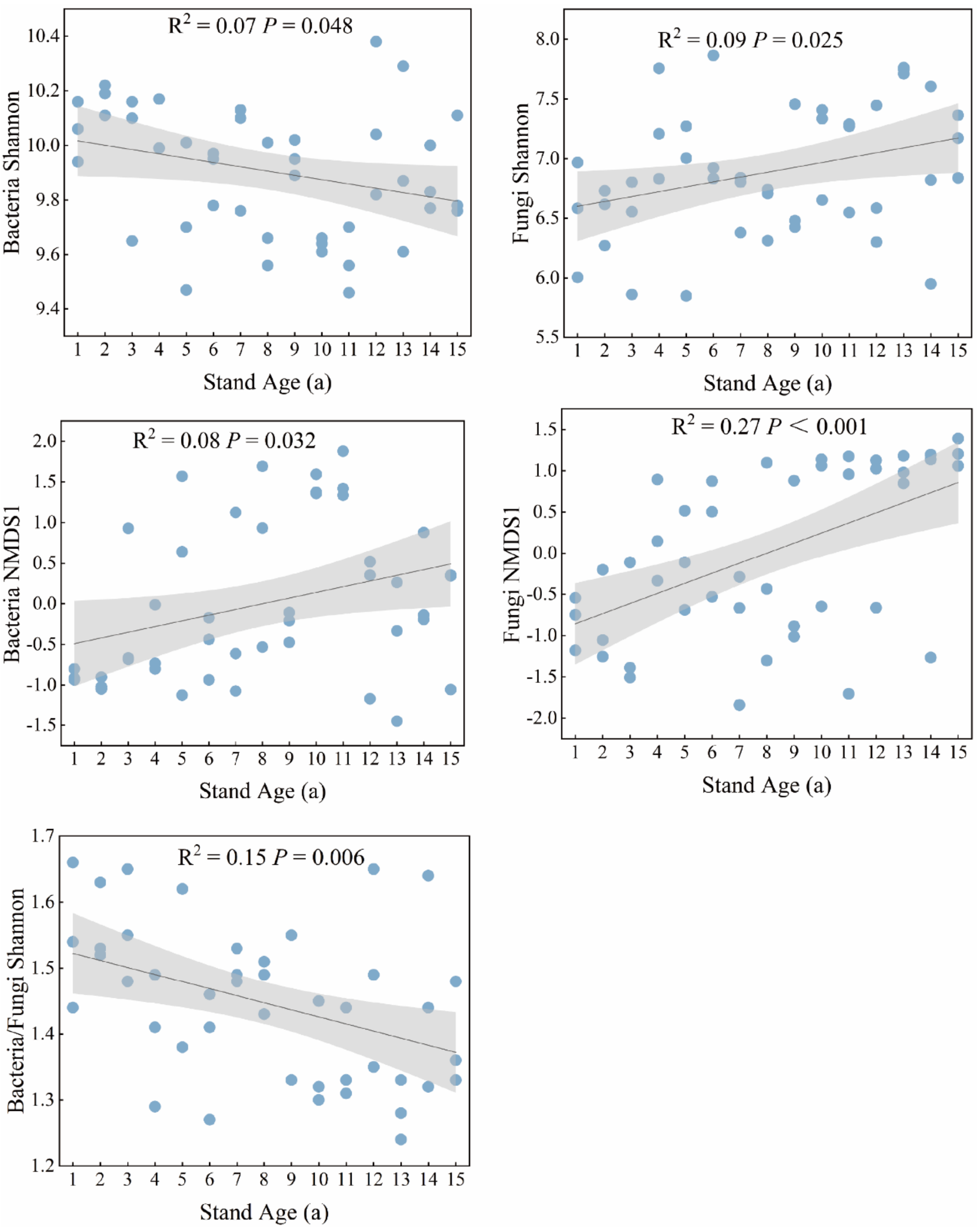

FIGURE S6. Relationship between microbial diversity and stand age. The solid black line indicates a significant linear relationship consistent with the regression model, and the shaded area indicates the fitted 95% confidence interval.

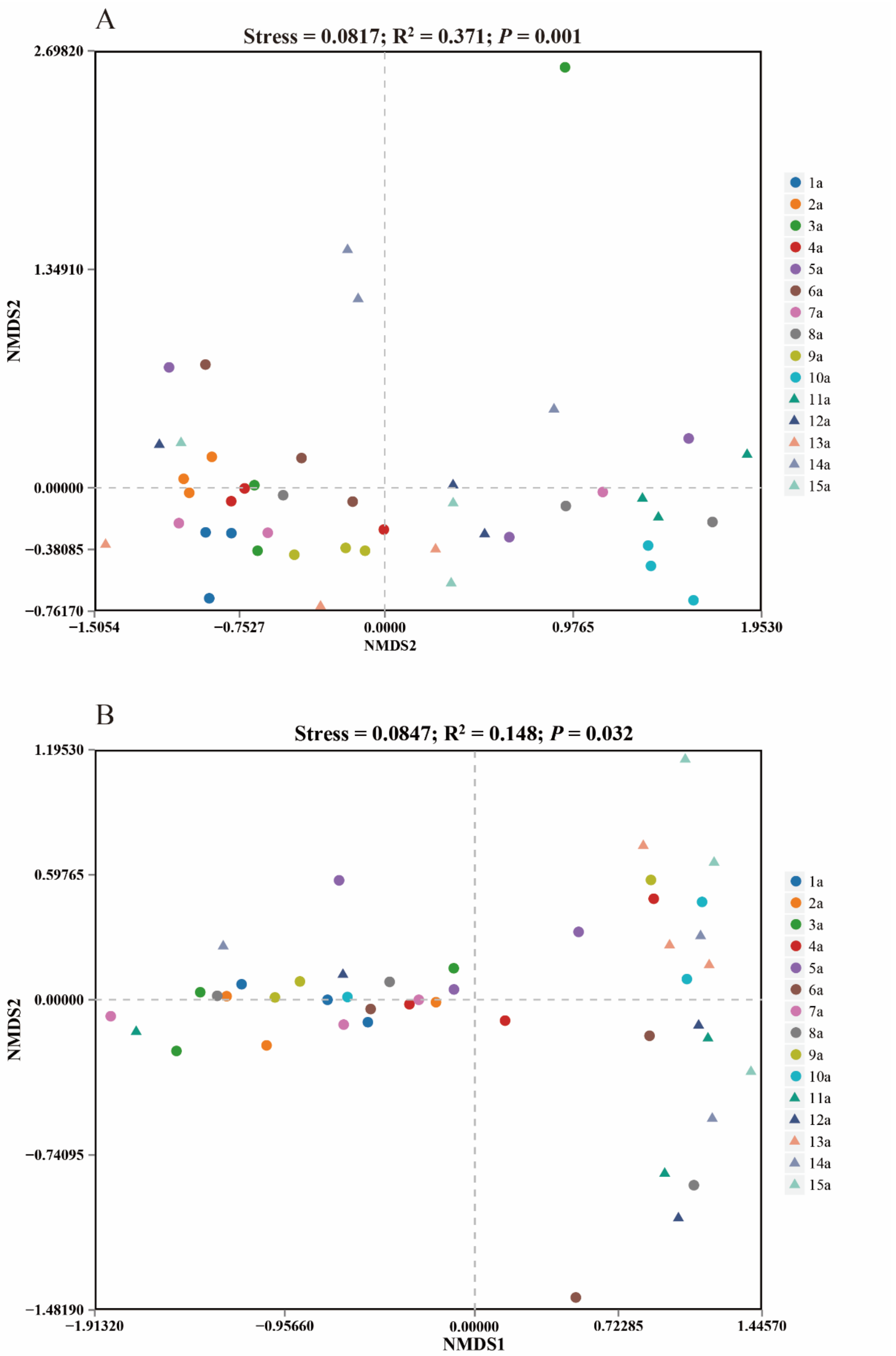

FIGURE S7. Non-metric multidimensional scale (NMDS) ordination based on Bray-Curtis similarities of bacterial (A) and fungal (B) communities on stand age.

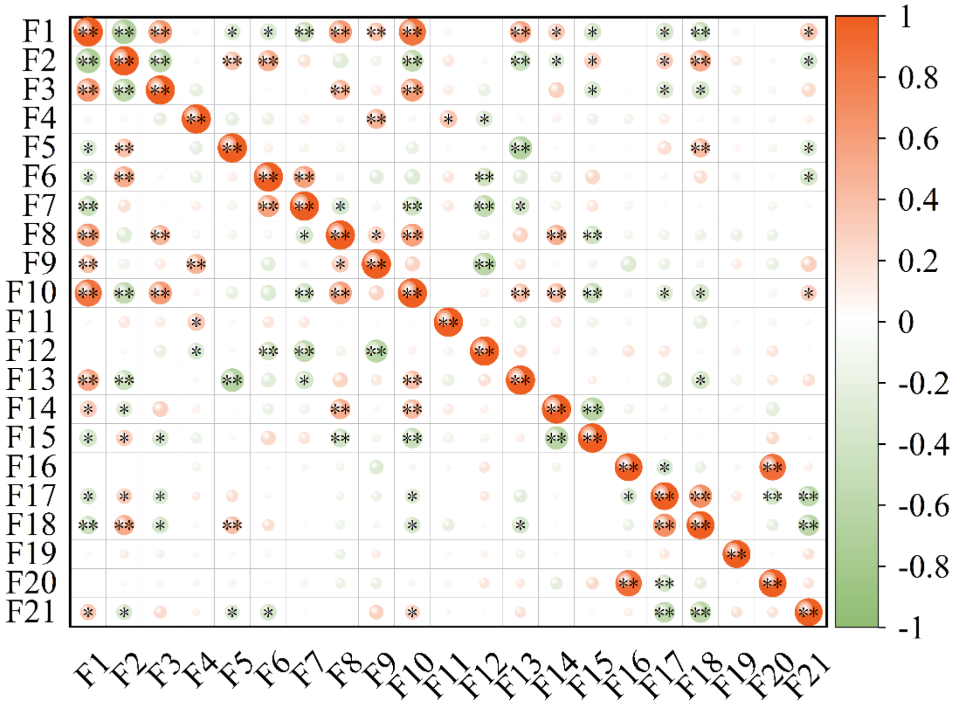

FIGURE S8. Pearson correlation analysis of soil respiration with biotic and abiotic factors in poplar plantation.Note:F1, Soil respiration; F2, Stand age; F3, Soil Temperature; F4, Soil Moisture; F5, Soil bulk density; F6, DOC/SOC; F7, EOC/SOC; F8, Available Potassium; F9, Total Nitrogen; F10, NO_3^--N contene; F11, NH_4^+-N contene; F12, Soil C/N; F13, Litter C/N; F14, Bacteria Shannon; F15, Bacteria NMDS1; F16, Bacteria NMDS2; F17, Fungi Shannon; F18, Fungi NMDS1; F19, Fungi NMDS2; F20, Bacterial Copiotrophic/oligotrophic; F21, Fungal Copiotrophic/oligotrophic. Statistically significant results ($P <= 0.05$) were labelled (* $P <= 0.05$; ** $P <= 0.01$).

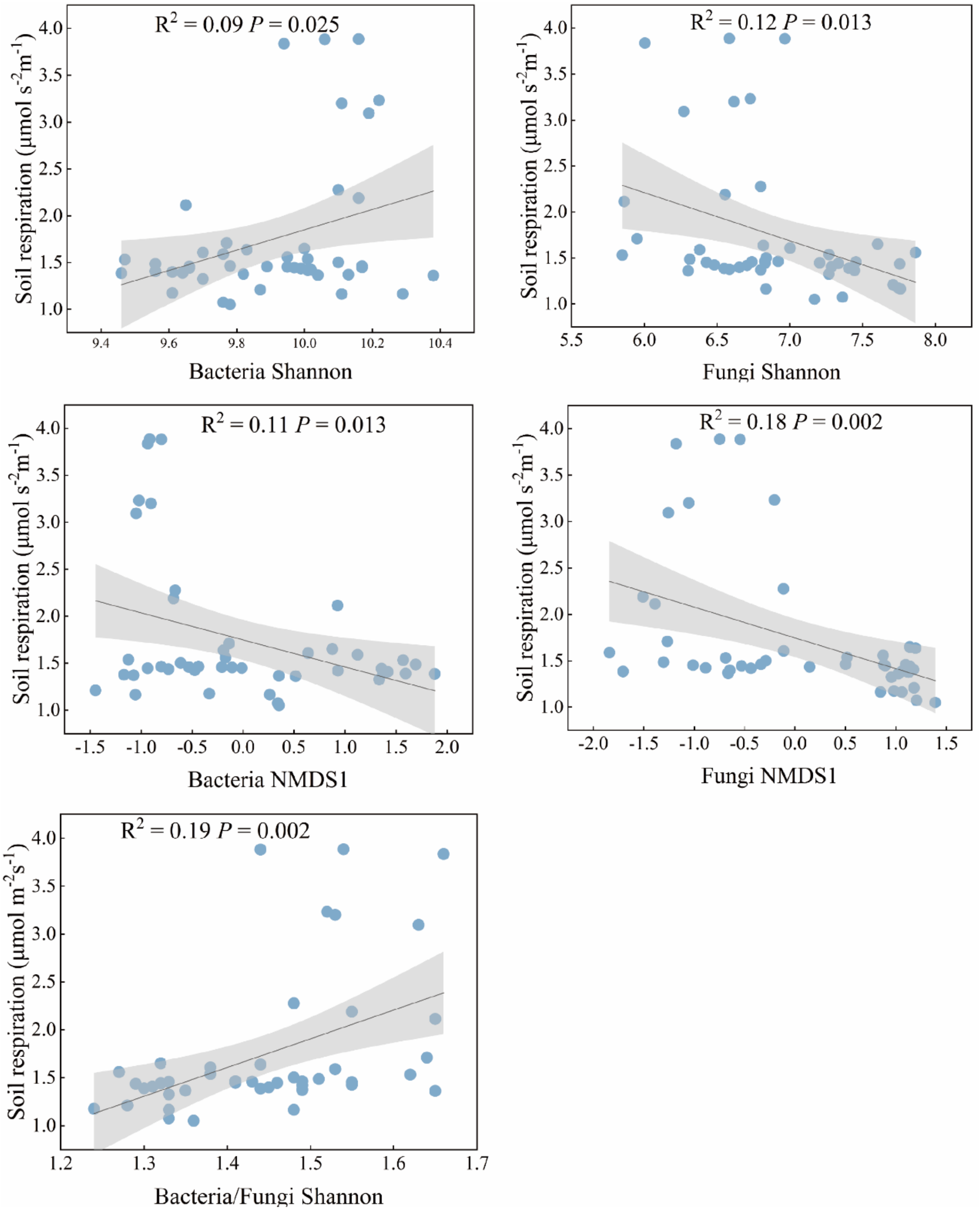

FIGURE S9. Relationship between microbial diversity and soil respiration. The solid black line indicates a significant linear relationship consistent with the regression model, and the shaded area indicates the fitted 95% confidence interval.
